# In silico analysis and in vivo assessment of a novel epitope-based vaccine candidate against uropathogenic *Escherichia coli*

**DOI:** 10.1038/s41598-020-73179-w

**Published:** 2020-10-01

**Authors:** Sara Hasanzadeh, Mehri Habibi, Mohammad Ali Shokrgozar, Reza Ahangari Cohan, Khadijeh Ahmadi, Mohammad Reza Asadi Karam, Saeid Bouzari

**Affiliations:** 1 Department of Molecular Biology, Pasteur Institute of Iran, Tehran, Iran; 2 National Cell Bank, Pasteur Institute of Iran, Tehran, Iran; 3 Department of Nanobiotechnology, Pasteur Institute of Iran, Tehran, Iran; 4 Infectious and Tropical Diseases Research Center, Hormozgan Health Institute, Hormozgan University of Medical Sciences, Bandar Abbas, Iran

**Keywords:** Biologics, Vaccines

## Abstract

Uropathogenic *Escherichia coli* (UPEC) are common pathogens in urinary tract infections (UTIs), which show resistance to antibiotics. Therefore, there is a need for a vaccine to reduce susceptibility to the infection. In the present study, bioinformatics approaches were employed to predict the best B and T-cell epitopes of UPEC virulence proteins to develop a multiepitope vaccine candidate against UPEC. Then, the efficacy of the candidate was studied with and without Freund adjuvant. Using bioinformatics methods, 3 epitope-rich domains of IutA and FimH antigens were selected to construct the fusion. Molecular docking and Molecular dynamics (MD) simulation were employed to investigate in silico interaction between designed vaccine and Toll-like receptor 4 (TLR4). Our results showed that the levels of IgG and IgA antibodies were improved in the serum and mucosal samples of the vaccinated mice, and the IgG responses were maintained for at least 6 months. The fusion protein was also able to enhance the level of cytokines IFN.γ (Th1), IL.4 (Th2), and IL.17. In challenge experiments, all vaccine combinations showed high potency in the protection of the urinary tract even after 6 months post first injection. The present study indicates that the designed candidate is able to evoke strong protective responses which warrant further studies.

## Introduction

Urinary tract infections (UTIs) are one of the most common infections occurring both in the community and hospitals, affecting 150 million people each year worldwide. UTIs are caused by different kinds of pathogens, but most commonly by uropathogenic *Escherichia coli* (UPEC)^1,2^. A urinary tract infection is a microbial invasion that results in an inflammatory response in the epithelium of the urinary tract^3^.

Adhesins and iron acquisition receptors are among the critical virulence factors of UPEC strains^1,2^. UPEC has several types of fimbriae, among which, type 1 pili and its critical adhesin, FimH, are necessary for the colonization, invasion, and protection of UPEC strains against the host defenses and antibiotics^4,5^. FimH has an N-terminal and a C-terminal domain, the N-terminal domain participates in the binding of UPEC to most host cell receptors^6^. Several studies revealed that the iron acquisition genes are among the highly expressed genes of UPEC strains during UTIs^7–9^. IutA is a siderophore receptor stable at low pH that is highly expressed. It has a high tendency to iron binding, and is significantly more prevalent among UPEC strains than among commensal serotypes^2,7,10^.

Antimicrobial therapy is the routine treatment for UTIs, but is suffers from an increasing rate of antimicrobial resistance among UPEC strains. Furthermore, UTI’s high incidence and significant costs emphasize the need for UTI vaccines^11,12^. To date, various studies have been conducted to develop an effective vaccine against UPEC, targeting virulence factors such as fimbriae and their adhesins (FimH, FimC, PapG, and PapD), toxins (Hlyα), and iron acquisition systems, including FyuA, IroN, chuA, IutA, and IreA^13–15^. Although there are several vaccine products available in some countries, there is yet no effective universal vaccine for the prevention or treatment of UTIs. However, there are several vaccines accessible in some countries. These vaccines include: (1) Uro-Vaxom, which contains extracts of 18 uropathogenic strains and is currently marketed in almost 40 countries worldwide, excluding the USA and Canada; (2) Solco-Urovac, a polymicrobial mixture of whole-cell and heat-killed uropathogens including six *E. coli* strains, and one strain of *Proteus mirabilis, Morganella morganii*, *Enterococcus faecalis* and *Klebsiella pneumoniae*; and (3) Urvakol/Urostim, a product that contains attenuated uropathogens strains of *E. coli*,* Proteus mirabilis*,* Enterococcus faecalis*, and *Klebsiella pneumoniae*^16^.

Due to the lack of an effective and universal vaccine against UTIs, there is a need to test different antigens and strategies to develop an ideal UTIs vaccine, especially against UPEC strains. The immune response to UTIs includes both innate and adaptive mechanisms^1^. UPEC strains are able to form intracellular bacterial communities (IBCs) in bladder epithelial cells that protect UPEC from immune responses and antibiotics^17^. Therefore, it is rational that both arms of immune responses, including humoral and cellular responses are taken into account for efficient eradication or prevention of UTIs caused by UPEC strains.

Recently, subunit vaccines, mostly consisting of MHC-I and MHC-II T-cell epitopes, and B-cell epitopes, have been considered^18^ because of their safety compared to killed and attenuated vaccines, their stability under different conditions, cost-effective production, high specificity, and capacity to deliver high doses of antigens^19,20^.

In the present study, with the aim of developing an effective candidate against UPEC, we used both computational approaches and experimental data to predict major histocompatibility complex (MHC) class I, MHC class II, and B cell epitopes in IutA and FimH antigens of UPEC strain CFT073. Moreover, all the selected epitope-rich domains were fused to each other by a pan HLA DR-binding epitope (PADRE), which is a helper epitope that binds to a broad range of mice and human MHC-II alleles and promotes T-helper cells^21,22^. Then, a 3D model of the candidate was docked with its cognate Toll-like receptor 4 (TLR4), and following a molecular dynamics (MD) procedure the binding affinity of the vaccine/receptor complex was evaluated^23^. Subsequently, the poly-epitope was expressed in *E. coli* BL21 (DE3) strain and purified using affinity chromatography. Finally, the immune responses induced by the vaccine administration with and without Freund adjuvant were investigated in mice and their protection efficacy evaluated in the urinary tract of the challenged mice.

## Result

### Immuno-informatics analyses

#### Defining linear B-cell epitopes

Since B-cell epitopes have an important role in humoral responses, full-length sequences of FimH and IutA were subjected to linear B-cell epitope prediction. Twenty mer epitopes with a cutoff more than 0.8 were selected using BCPred and IEDB servers. IEDB showed several continuous predicted epitopes in both FimH and IutA proteins. Therefore, the regions containing the highest number of epitopes predicted by two servers were selected to increase the accuracy of prediction (Table 1).

**Table 1:** Final linear B-cell epitopes selected from full-length proteins FimH and IutA using BCPred and IEDB.

Antigen	Amino acid position	Sequences
IutA	336–369	LVGQVYYRDESLRFYPFPTVNANKQATAFSSSQ
	365–377	FSSSQQDTDQYGMK
	351–374	PFPTVNANKQATAFSSSQQDTDQY
	456–476	GGVRYQYTENRVDDFIDYTQ
	481–501	AGKAISADAIPGGSVDYDNF
	420–437	NNHKIYTTGRYPSYDIT
	526–545	LPDPGKYYGRGIYGAAVNG
	561–590	AGTKSGFNSSKDHDERIAGAVSGGNDHISG
FimH	25–45	KTANGTAIPIGGGSANVYVN
	46–66	APAVNVGQNLVVDLSTQIFC
	68–89	DYPETITDYVTLQRGSAYGGV
	110–133	ETPRVVYNSRTDKPWPVALYLTP
	90–125	TVKYNGSSYPFPTTSETPRVVYNSRTD
	120–150	DKPWPVALYLTPVSSAGGVAIKAGSLIAVL

#### Defining T-cell epitopes

According to multiple alleles of MHC-I and II, high score predicted epitopes were determined for both proteins. Sixteen 9-mer epitopes were predicted as MHC-I binders, and 21 epitopes with high binding affinity score of < 50 IC_50_ nM were predicted as MHC-II binders. The selected human and mouse MHC-I and MHC-II epitopes are summarized in Table 2 and the output data of each tool are provided in Supplementary Table S1–S4.

**Table 2:** Final MHCI and MHCII binding regions selected from FimH and IutA.

	MHC-I		MHC-II	
	Human	Mouse	Human	Mouse
FimH positions	88–96 141–152	41–49 122–130 127–135 144–152	36–54 49–66 70–90 108–126 124–138 141–160	35–50 42–64 82–107 107–121 122–142 140–154
IutA positions	355–369 499–507	402–411 430–438 458–466 499–505 511–518 545–553 585–593	335–349 581–599 599–613	355–369 473–487 481–495 513–549 580–599 598–612

#### Designing of epitope-rich domains for final vaccine construct

**Figure 1:**
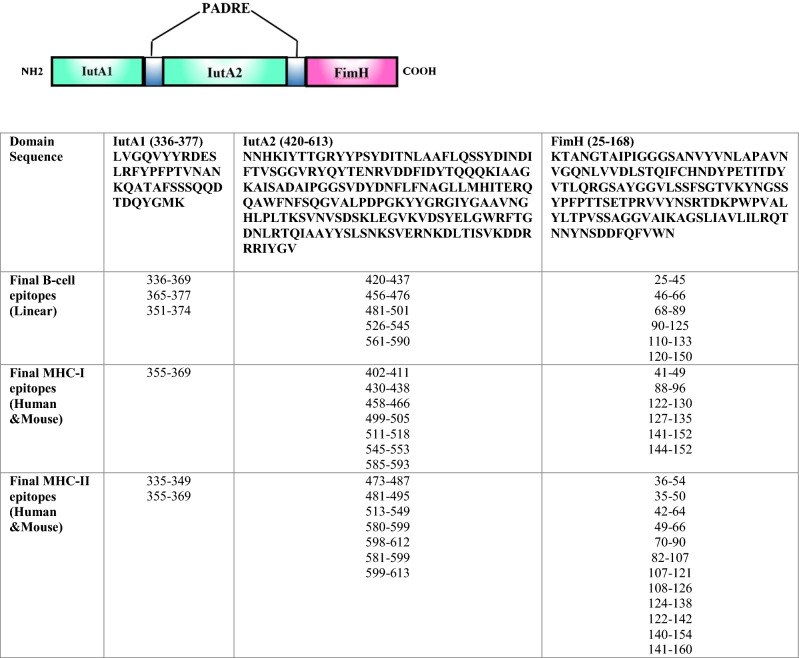
Details of the domain sequences and B- and T- cell epitopes of IutA and FimH in the final construct linked together with PADRE linkers.

Based on immuno-informatics analysis, B-cell, MHC-I, and MHC-II epitopes with the highest scores were selected. The final vaccine construct was made with two domains of IutA (336–377 and 420–613) and the N-terminal domain of FimH (25–168). The domains were joined together by PADRE sequence as a linker. The details of the selected linear B- and T- cell epitopes in the final construct are shown in Fig. 1.

### Vaccine features

#### Assessment of the properties of the designed sequence

Based on VaxiJen v2.0 server, the antigenicity value of the sequence was estimated to be 55% at a threshold of 0.4%. Furthermore, the final sequence was predicted as a non-allergenic sequence on AlgPred. According to the results of SOLpro, the solubility of the sequence upon overexpression in *E. coli* was 0.65%. The results of ProtParam indicated that the molecular weight (Mw) and theoretical isoelectric point value (pI) of the protein were 45.7 kDa and 8.7, respectively. Aliphatic index (77.75) and obtained grand average of hydropathicity (GRAVY) (− 0.302) values elucidated the hydrophilicity of the designed sequence. Finally, the designed sequence was predicted as a stable protein by calculation of instability index (31.73) using the ProtParam.

#### 3D structure prediction and validation

**Figure 2:**
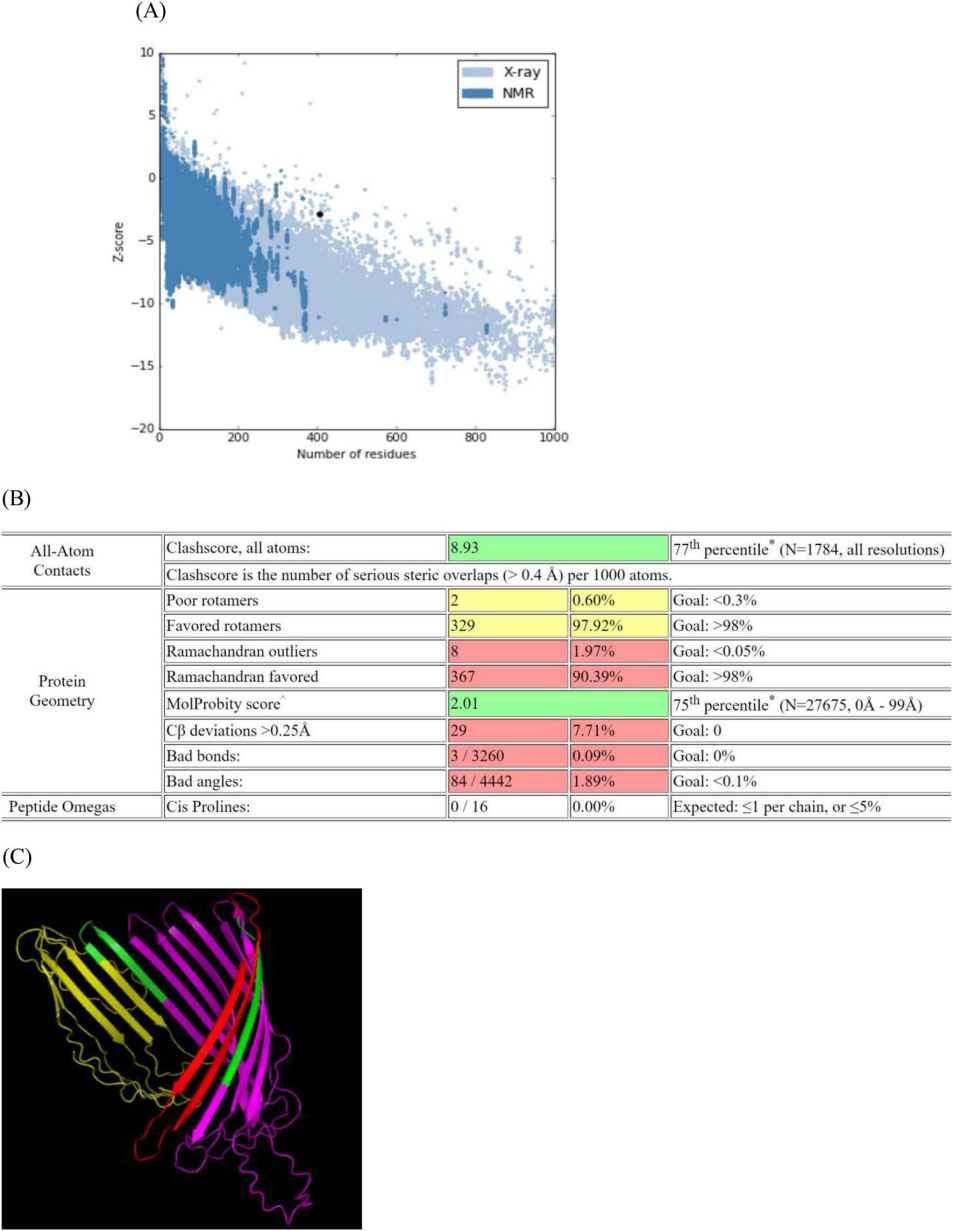
Validation and prediction of refined 3D model of protein. (**A**) Z-score of this model is − 2.8, that is in the range of native protein conformation. (**B**) Molprobity result of the refined construct. (**C**) Predicted 3D model of the final construct was visualized by PyMOL Viewer tool (The PyMOL Molecular Graphics System, Version 1.1, Schrödinger, LLC). The FimH, IutA1, and IutA2 domains, and linkers are displayed in red, violet, yellow, and green colors, respectively.

Five 3D models of the designed vaccine were modeled by I-TASSER, and those with maximum C-score were selected. Template modeling score (TM score) of the best model was 0.74 ± 0.15 that indicated the structural similarity based on the structural alignment. According to ProSA, Z-score of the selected model was − 2.02, which was in the range of scores for native proteins with similar sizes (Fig. 2). Ramachandran plot showed that 71.6% of residues were located in the favored regions. Refinement of the predicted structure improved the allowed amino acids, and Z-score of the refined model to 92.30% and − 2.8, respectively. In Molprobity analysis, all-atom clash and Molprobity scores were calculated to be 8.93 and 2.01, respectively. In addition, the expected RMSD was calculated to be 3.51 by TM-align (Fig. 2B). The 3D model of final construct was visualized by PyMOL Viewer (Fig. 2C).

#### Prediction of discontinuous B-cell epitopes

According to the important role of conformational epitopes in humoral response, conformational B cell epitopes of the designed sequence were predicted on the basis of protein-antibody interaction. Discontinuous peptides with a score value of 0.7 or more were selected, and the scores showed surface protein atoms responsible for binding to antibodies. The compositions of amino acids, the number of amino acids, sequence location, as well as the score values are summarized in Table 3. Furthermore, 3D representation of the predicted discontinuous epitopes of the final protein is shown in Supplementary Fig. S1.

**Table 3:** Conformational epitopes of the designed protein as predicted by ElliPro.

No.	Residues	Number of residues	Score
1	A:Y7, A:D9, A:E10, A:S11, A:L12, A:R13, A:F14, A:P16, A:F17, A:P18, A:T19, A:V20, A:N21, A:A22, A:N23, A:Q25, A:N57, A:H58, A:K59, A:I60, A:Y61, A:T62, A:T63, A:G64, A:R65, A:Y66, A:Y67, A:P68, A:D105, A:D106, A:F107, A:I108, A:D109, A:Y110, A:T111, A:Q112, A:Q113, A:Q114, A:K115, A:I116, A:A117, A:A118, A:G119, A:K120, A:A121, A:I122, A:S123, A:A124, A:D125, A:A126, A:I127, A:P128	52	0.752
2	A:T258, A:L259, A:K260, A:A261, A:A262, A:V295, A:V296, A:D297, A:L298, A:S299, A:T300, A:Q301, A:I302, A:F303, A:R320, A:G321, A:S322, A:A323, A:Y324, A:G325, A:G326, A:V327, A:L328, A:S329, A:S330, A:F331, A:S332, A:K361, A:P362, A:W363, A:P364, A:V365, A:A366, A:L367, A:Y368, A:L369, A:T370, A:P371, A:V372, A:S373, A:S374, A:A375, A:G376, A:G377, A:V378, A:A379, A:I380, A:K381, A:A382, A:G383, A:S384, A:L385, A:I386, A:A387, A:V388, A:V405, A:W406, A:N407	58	0.745
3	A:L1, A:V2, A:G3, A:Q4, A:V5, A:F29, A:S31, A:S32, A:Q33, A:Q34, A:D35, A:T36, A:D37, A:Q38, A:Y39, A:G40, A:M41, A:K42, A:A43, A:K44, A:F45, A:V46, A:A47, A:A48, A:W49, A:L79, A:S81, A:S82, A:Y83, A:D84, A:I85, A:N86, A:D87, A:I88, A:F89, A:T90, A:V91, A:G93, A:V95, A:Y97, A:L143, A:L144, A:M145, A:H146, A:I147, A:T148, A:E149, A:R150, A:Q151, A:A153, A:W208, A:G212, A:D213, A:N214, A:L215	55	0.735

#### Molecular docking of TLR4-protein vaccine

**Figure 3:**
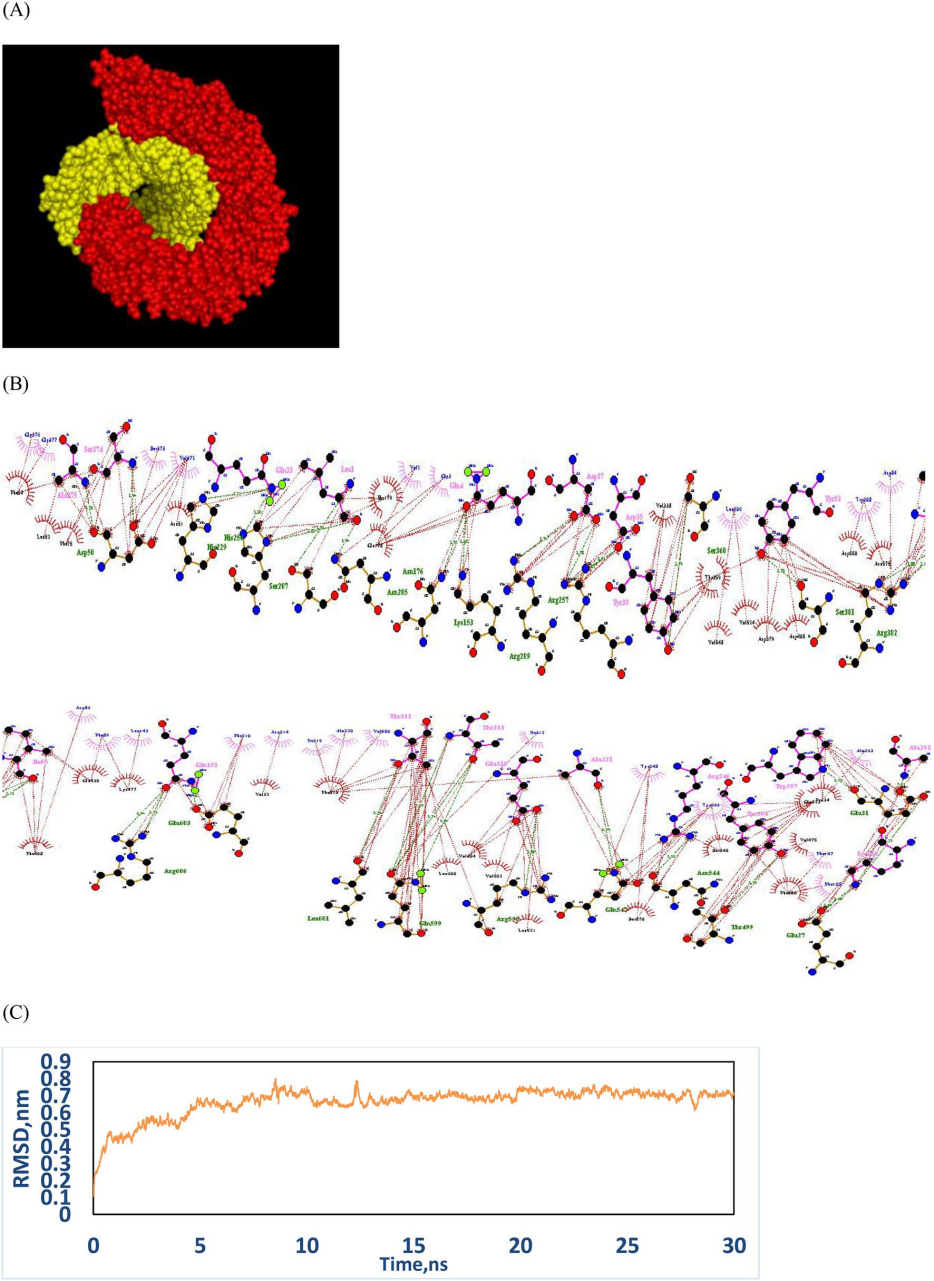
(**A**) Docking model of the designed construct and TLR4. TLR4 is in red and protein vaccine is in yellow. (**B**) Hydrophobic interactions and hydrogen bonding between TLR4 and vaccine. Hydrogen bonds are shown by blue dashed lines between TLR4 (green) and construct (pink) residues, and hydrophobic interactions are shown by red dashed line between spoked arcs representing residues of TLR4 (black) and construct (blue). (**C**) Backbone RMSD plots of the vaccine candidate in complex with TLR4.

The best docked model was selected based on the total free energy (− 1829.9) and best interaction tendency to TLR4 (Fig. 3A). Hydrophobic interactions, as an important factor for initiating innate immune responses, were dominant signs for the interaction between TLR4 and the designed vaccine (Fig. 3B).

#### Molecular dynamic simulations

The molecular dynamics (MD) simulation showed that the potential energy of the simulated system remained stable during the simulation time. The RMSD plots for TLR4 and the designed construct are shown in Fig. 3C and confirmed the stability of the designed construct in interaction with TLR4.

#### Sequence analyses

The results of homology alignment of the selected epitopes in FimH and IutA with those deposited in National Centre for Biotechnology Information (NCBI) showed that these sequences were conserved among UPEC strains (> 97% for FimH and > 90% for IutA) (Supplementary Figs. S2 and S3).

#### Codon optimization and cloning

**Figure 4:**
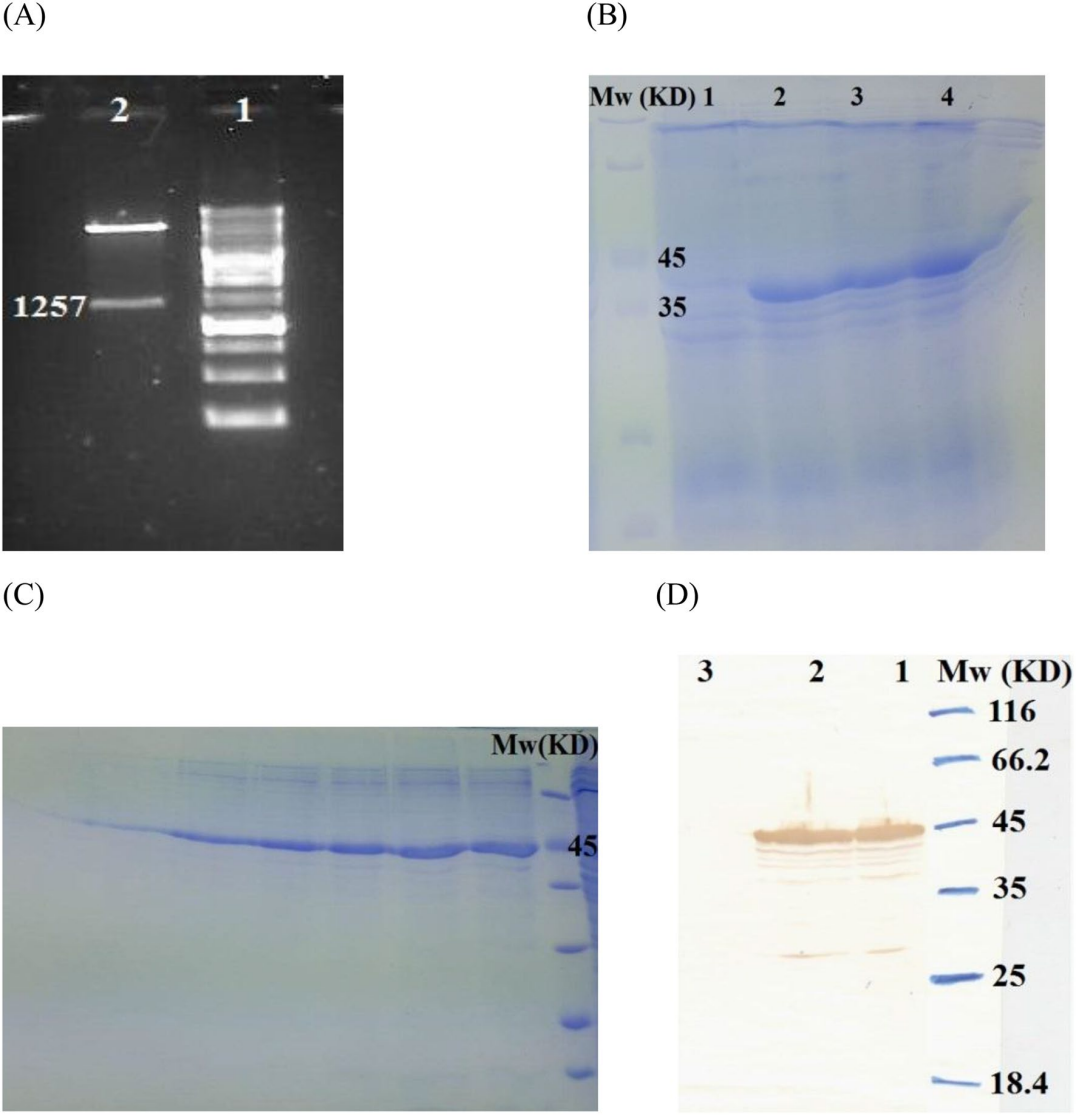
(**A**) Digestion of the cloned gene in pET28a vector by *Nco*I and *Hind*III enzymes (lane 1: 1 kb Mw and lane 2: digested pET28a-gene). (**B**) SDS-PAGE (lane 1: Negative control and lane 2–4: designed protein (∼ 45 kDa) in 0.5, 0.75, and 1 mM IPTG concentration). (**C**) Purification of the expressed protein in different elutes, and (**D**) Western blot of the expressed protein (lane 1 and 2: designed protein (∼ 45 kDa) in 0.75 and 1 mM concentrations of IPTG and lane 3: Negative control).

The codon optimization of the DNA sequence to the codon usage of *E. coli* was carried out by Biomatik Company and OPTIMIZER server. The gene was cloned into pET28a expression vector using *Nco*I and *Hind*III restriction sites with a poly histidine-tag (6x-His tag) at C-terminus of the protein. The fidelity of cloning was finally verified by gel electrophoresis, PCR, restriction map analysis (Fig. 4A), and sequencing.

#### Purification and confirmation of the recombinant protein

The protein expression was confirmed by SDS-PAGE and Western blot. The SDS-PAGE (Fig. 4B) and Western blot showed a 45 kDa band as expected by Mw calculations. The recombinant protein was purified from *E. coli* DE3 lysates by applying onto Ni–NTA affinity column. The purified protein was verified by SDS-PAGE (Fig. 4C) and Western blot analysis (Fig. 4D). The LAL test indicated a low amount of LPS (< 0.5 EU/ml) in the protein solution.

#### Determination of IgG responses in human cases

**Figure 5:**
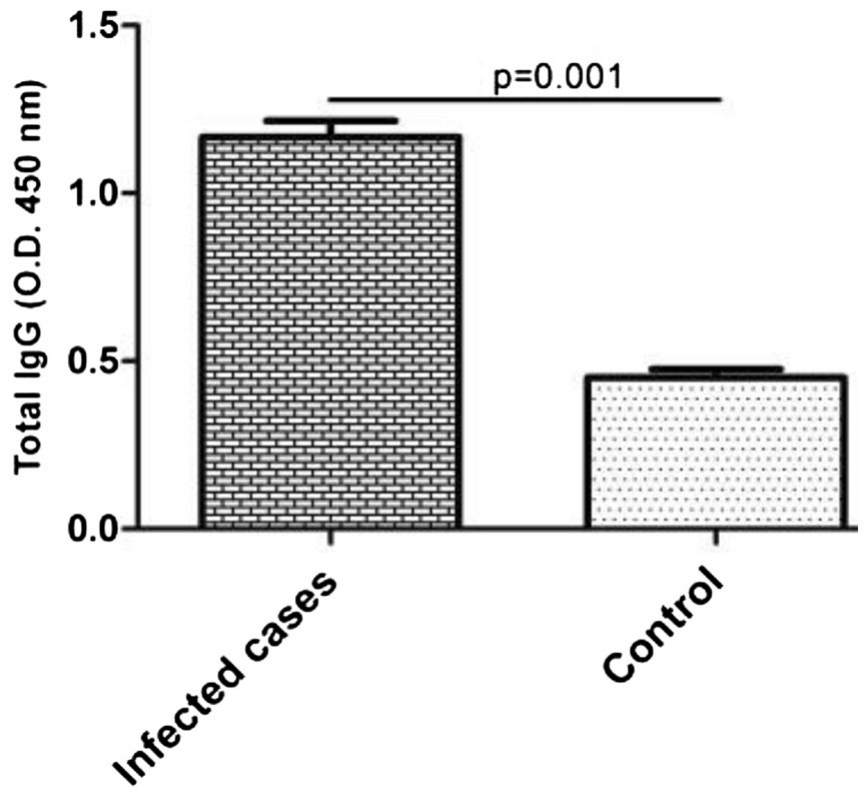
Evaluation of the humoral responses in human cases. The levels of IgG were measured in humans who had been previously infected with UPEC and the results showed a statistically significant higher level of IgG as compared to the control (p < 0.05). Bars represent mean ± S.D. from 10 cases per groups. Statistical analysis was performed using Student t-test.

The levels of IgG response against the recombinantly produced protein construct were measured in humans who had been previously infected with a pathogenic UPEC. Those infected showed a statistically significant higher level of IgG as compared to the humans that had no history of infection with a UPEC strain (p < 0.05) (Fig. 5).

#### Humoral immune responses in the immunized mice

**Figure 6:**
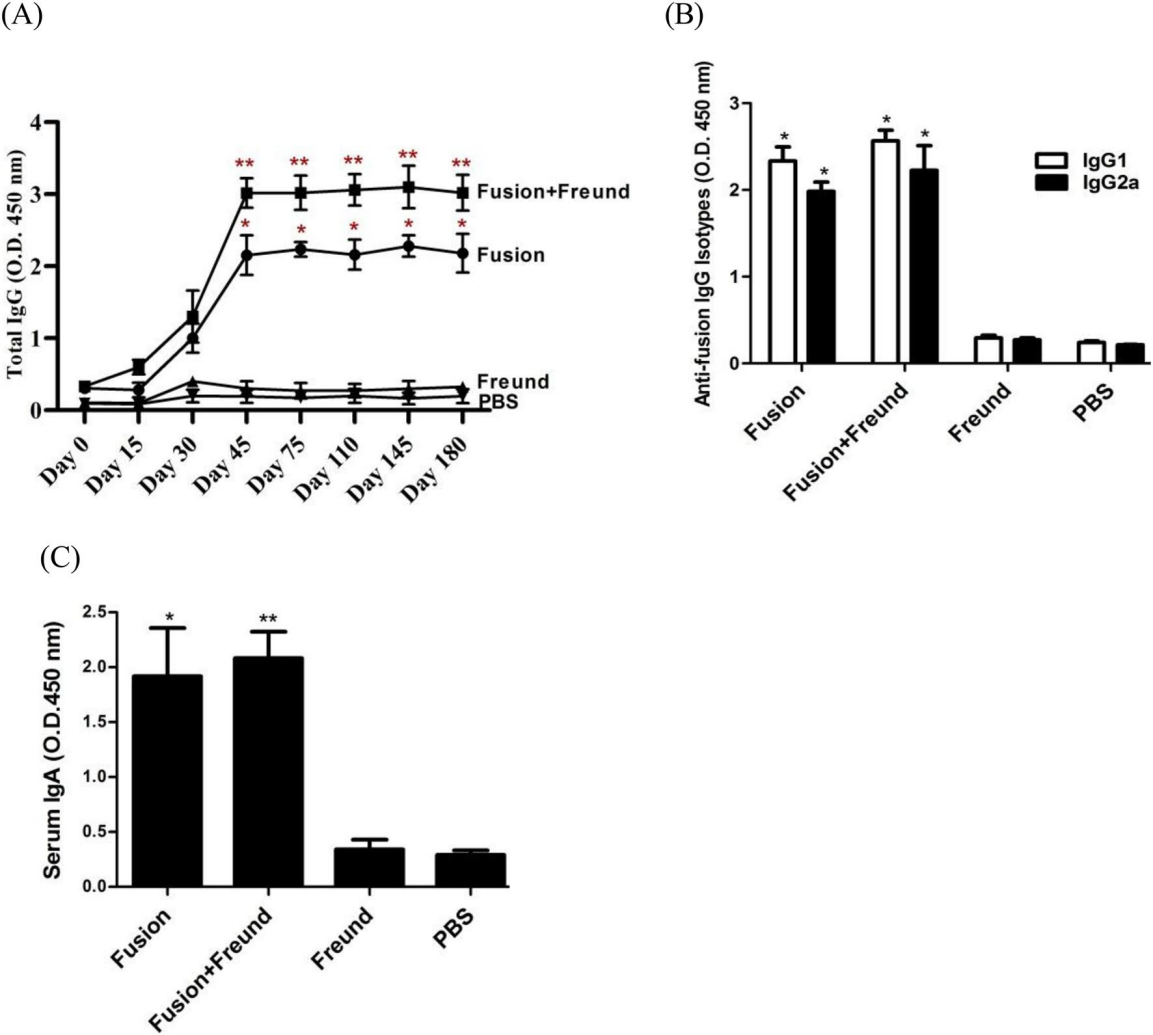
Evaluation of the humoral responses in the immunized mice. (**A**) Mice were vaccinated and the IgG responses and longevity of humoral responses measured. Single asterisks show statistical significance of IgG responses over the control groups from day 45 to 180 post first vaccine inoculation (p < 0.001), and double asterisks show statistical significance of IgG over fusion protein alone (p < 0.05) and control groups (p < 0.001). The results are the average of three independent experiments at serum dilution 1:800. Then, (**B**) anti-fusion IgG1 & IgG2a and (**C**) IgA responses were assessed in the mice. Single asterisks show statistical significance of IgG1 & IgG2a and IgA responses over the control groups (p < 0.001), and double asterisk shows statistical significance of IgA over fusion protein alone (p = 0.045). The results are the average of three independent experiments at dilution 1:200 serum. Bars represent mean ± S.D. from 12 mice per groups. Statistical analysis of total IgG, IgG isotypes, and IgA responses was performed using One way ANOVA test.

The mice groups that received fusion protein alone or with Freund adjuvant showed higher IgG responses compared to the control groups that received just PBS or Freund. Moreover, data analysis indicated that adding the Freund to fusion protein resulted in a statistically significant higher IgG response than inoculating the protein alone (Fig. 6A).

The evaluation of longevity of humoral responses revealed that mice vaccinated with fusion protein, and fusion plus Freund could significantly enhance IgG responses after the second immunization until day 45, and the immune responses they had induced remained steady up until 6 months post first vaccination. Overall, IgG responses induced by fusion and fusion plus Freund were significantly higher than those in control groups in all weeks. Moreover, the induced IgG response by fusion and fusion plus Freund was statistically different throughout the six months after the first vaccination (Fig. 6A).

#### Measurement of antibody isotype responses

Levels of systemic isotype antibodies (IgG1, IgG2a, and IgA) were measured after the third vaccination dose in mice. All mice raised significant levels of IgG1, IgG2a, and IgA against fusion protein compared to the control groups. Addition of Freund adjuvant to the fusion protein could not significantly improve the levels of IgG1 and IgG2a, whereas it raised the IgA levels in a statistically significant manner compared to the administration of fusion protein alone (Fig. 6B,C).

#### Mucosal antibody responses

**Figure 7:**
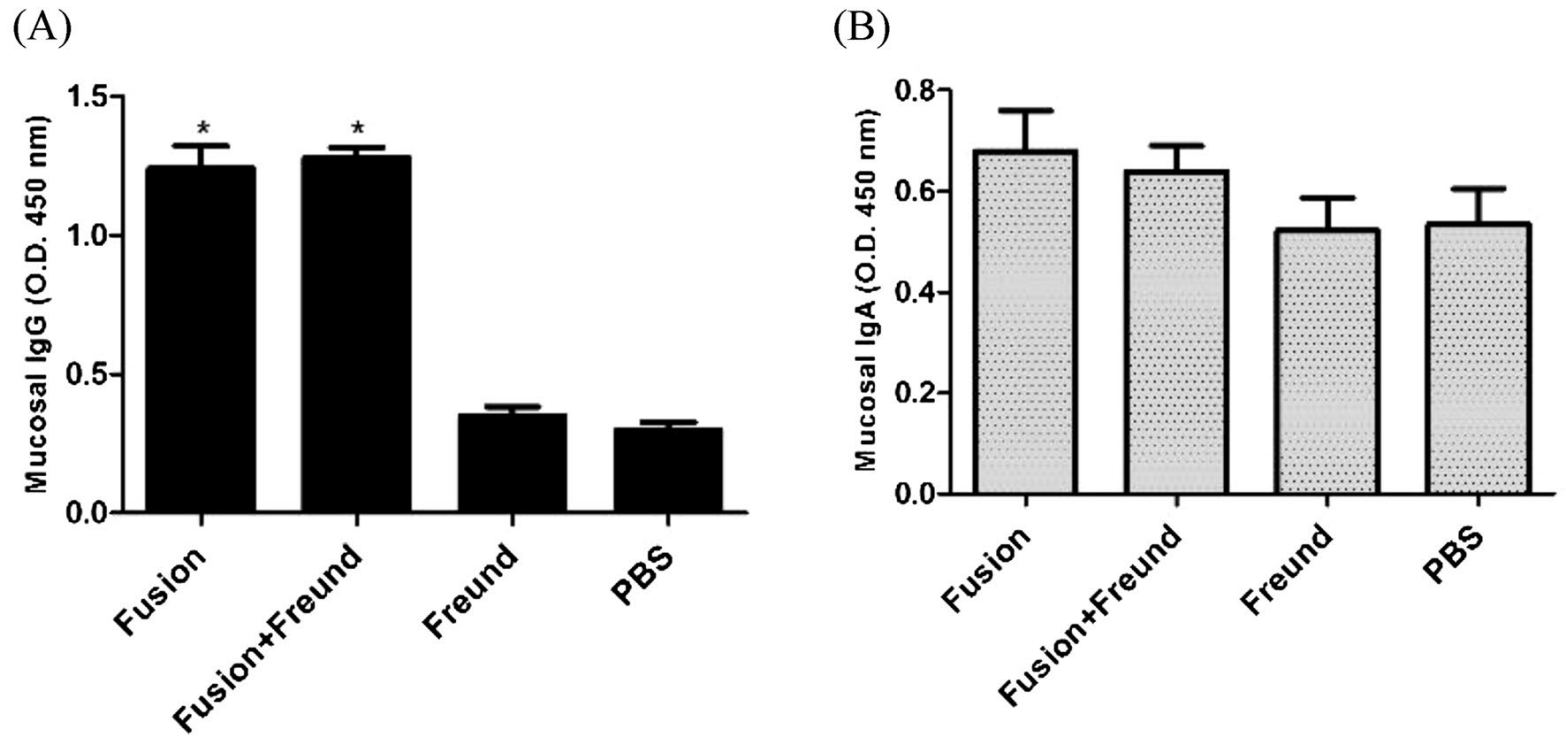
Measurement of mucosal responses. (**A**) Mucosal IgG and (**B**) IgA levels were measured in the urine. Single asterisks represent statistical significance of the combinations over the control groups (p < 0.05). The results are the average of three independent experiments. Bars represent mean ± SD from 12 mice per groups at dilution 1:5 of the urine samples.

Mucosal responses were determined in urine samples collected 2 weeks after the last vaccination. According to the results shown in Fig. 7, mice vaccinated with both fusion and fusion plus Freund could enhance the IgG levels in comparison with the control groups. We found that Freund adjuvant could not significantly raise the IgG level of the fusion protein as compared to the fusion alone group. It was also observed that the vaccine formulations, including fusion and fusion plus Freund could not significantly enhance the mucosal IgA levels when compared to the control groups (Fig. 7).

#### Measurement of cytokine responses

**Figure 8:**
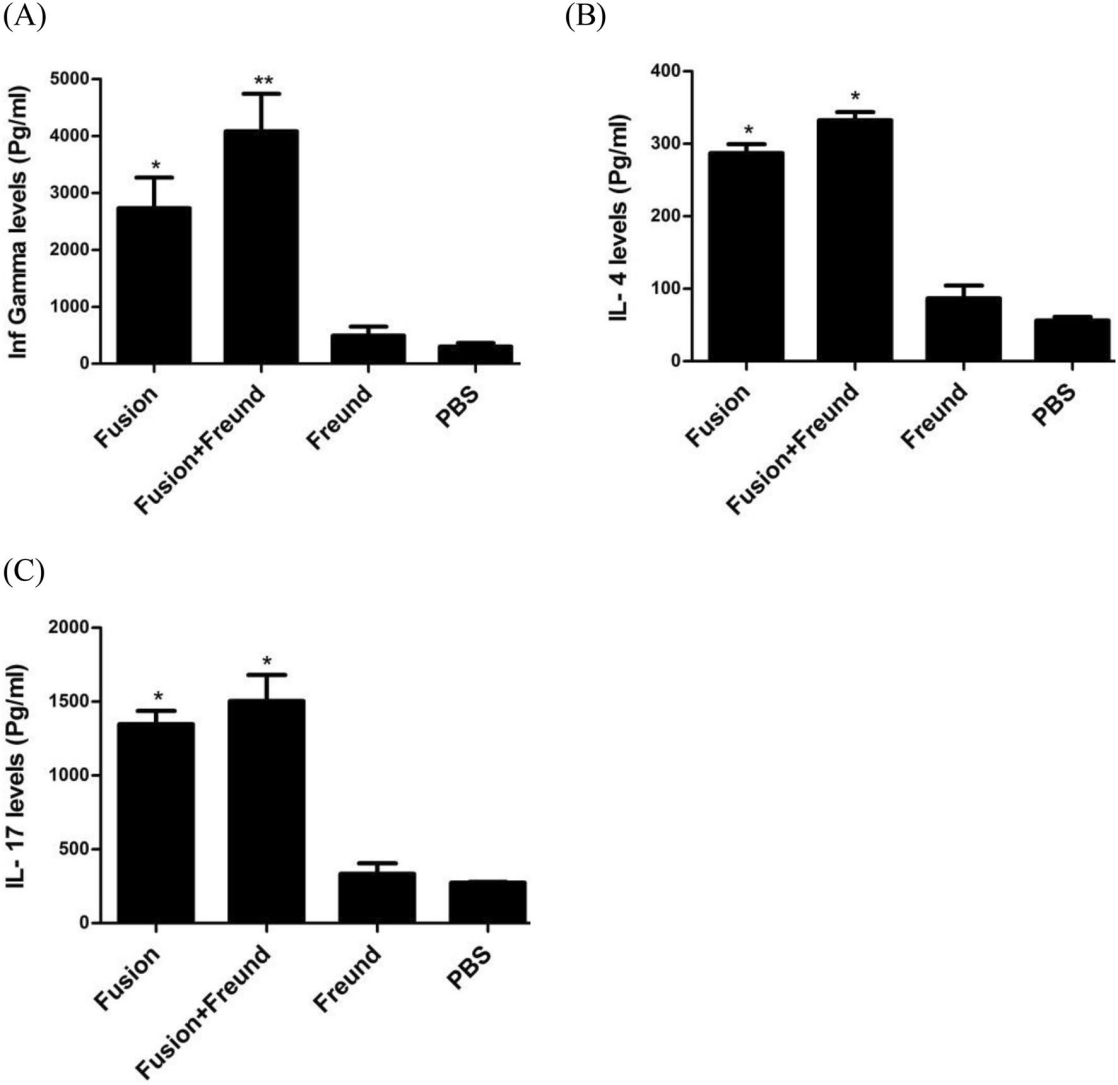
Measurement of the cytokine levels in the mice. The splenocytes of mice were stimulated with the fusion protein and analyzed for production of (**A**) IFN.γ, (**B**) IL.4, and (**C**) IL.17. The single asterisks show statistical significance of IFN.γ, IL.4, and IL.17 over control groups (p < 0.001). Double asterisk indicates statistical significance of IFN.γ levels over the non-adjuvant combinations and control groups (p < 0.001). Results are the mean stimulation index ± S.D. of five mice per group from three independent experiments.

According to the ELISA data, higher levels of IFN.γ, IL.4, and IL.17 cytokines were measured in supernatant of the splenocytes isolated from immunized groups as compared to control groups (p < 0.001). These findings showed that immunization with fusion plus Freund significantly increased the IFN.γ levels compared to the fusion protein alone (p < 0.001). While there was no statistical significant difference between the production of IL.4 and IL.17 in mice immunized with the fusion plus Freund, and the fusion alone (Fig. 8).

#### Vaccine efficacy against kidney and bladder challenge

**Figure 9:**
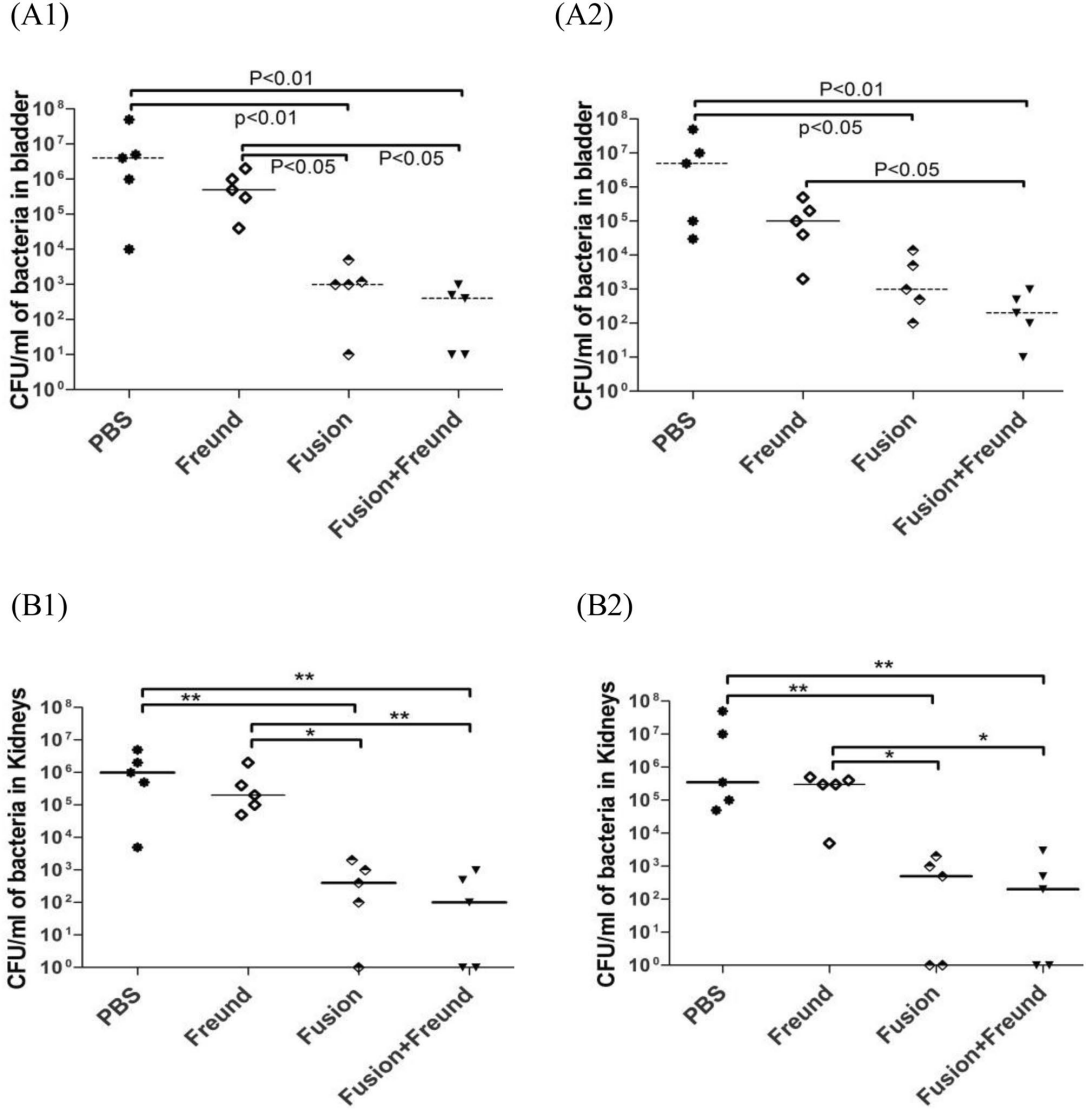
Results of bladder challenge against UPEC. On days 48 and 180 after the first vaccination, the bladders of mice (n = 5 for each time) were infected with *E. coli* CFT073. Seven days post challenge, the loads of UPEC were determined in the bladders (**A1**: day 48 and **A2**: day 180) and kidneys (**B1**: day 48 and **B2**: day 180) of the mice. Solid lines indicate median of the colonization levels. Statistical significance of the differences between the mice groups were determined by Kruskal–Wallis analysis (Dunn’s multiple comparison test) and are shown in between brackets with asterisks or P values. The single asterisk shows statistical significance of control mice over vaccine combinations (p < 0.05). Double asterisks indicate statistical significance of control mice over vaccine formulations (p < 0.01).

Challenge experiments were performed using UPEC strain CFT073 48 and 180 days after the first vaccination on the mice that were immunized with three doses. As a result of the first challenge, the mice groups immunized with the fusion or the fusion plus Freund showed a significant reduction (10^2^–10^3^ folds) in bacterial loads in both bladder and kidney organs as compared to the control groups (p < 0.05) (Fig. 9A1,B1). We also observed that there was no significant decrease in the levels of UPEC in the bladder and kidney of the mice that received the fusion alone and the fusion plus Freund. In addition, the results of the challenge after 6 months indicated that the bacterial levels in the vaccinated groups were significantly reduced (10^2^–10^3^ folds) in the bladder (p < 0.01) and kidneys (p < 0.01) as compared to the control groups (Fig. 9A2,B2).

## Discussion

Immunotherapy is the most potent way to prevent infectious diseases such as UTIs caused by UPEC strains. Safety, immunogenicity, and induction of protective immunity against a broad spectrum of UPEC strains are the ideal criteria of a vaccine against UPEC^24^.

Bioinformatics programs could define immune dominant B and T cell epitopes of antigens that play important roles in pathogenicity, and induction of immune responses^25,26^. The present study aimed to design and construct a novel multi-epitope protein from UPEC strain based on in silico methods to evoke protective immune responses.

Along with all the positive features of poly epitope vaccines, the main problem is their low immunogenicity. The usage of epitope rich domains is one of the strategies to overcome this problem which was applied in this construct^15^. These domains could induce robust T and B-cell immune responses without having the limitations of single epitopes^15^. Due to the importance of T-cell mediated immunity in the levels of B-cell responses in the protection against UPEC infections^17^, we decided to include MHC-I epitopes in the construct together with the MHC-II epitopes.

Given the importance of linkers in designing of vaccine candidates, we decided to apply a peptide epitope for T-helper cells instead of routine linkers, derived from HLA PADRE. In fact, PADRE has the capacity to evoke effective CD4 + T cell responses in inducing both the humoral and cellular responses^27,28^. Previous studies showed that FimH could induce innate host responses by interaction with TLR4 expressed on immune cells^6,23^. Toll-like receptors are among the pattern recognition receptors (PRRs) that can induce different aspects of immune responses such as secretion of pro-inflammatory cytokines^29^. Although the best linear B- and T-cell epitopes of FimH were located in amino acids 25–160 (Tables 1, 2), the results of defining the conformational epitopes showed that amino acids 166–168 of FimH were also among the selected discontinuous epitopes. Furthermore, the construct composed of amino acids 25–168 in FimH showed better confidence score and stability than the construct made of residues 25–160 in FimH. Finally, based on the linear and conformational epitopes and also evaluation of structures, we decided to design a construct which composed of two domains of IutA (336–377 and 420–613) and the N-terminal domain of FimH (25–168). According to our results, our designed vaccine could maintain sustainable interaction with TLR4 to have the chance to induce the humoral and cellular responses.

An ideal vaccine against UPEC should be widely distributed among different pathogenic strains and possess epitopes that are conserved among the strains. Therefore, the amino acid sequences of FimH and IutA in UPEC CFT073 strain used for construction of the multi-epitope were compared with the corresponding sequences deposited in NCBI, revealing that the sequences were > 90% identical. This comparison revealed the conservation of the selected epitopes of FimH and IutA among different UPEC strains; therefore the use of a single UPEC strain (UPEC CFT073) in this study can be attributed to the other UPEC strains.

It was found that the humans who were infected with the UTIs caused by UPEC strains developed humoral response against the vaccine candidate. The fact that humans infected by UPECs managed to respond to the vaccine protein showed that the predicted B cell epitopes, at least some of them, were indeed identified by clinical samples validating the predictions.

The use of a proper adjuvant is another strategy to promote the immune responses and direct them to a desired direction^30^. We decided to use a well-established adjuvant such as Freund to examine how much it improved the immunogenicity of the designed candidate. Freund has been used as one the most effective adjuvants for production of high titers of antibodies^31^, and induction of cell-mediated immunity (Th1)^32^. Our findings showed that the fusion protein without adjuvant could evoke significant humoral (IgA, IgG1, and IgG2a) and cellular (IFN.γ and IL.4) responses, demonstrating the ability of the candidate in raising a mixed response of type 1 (Th1) and type 2 (Th2). Freund could significantly increase the levels of total IgG and IFN.γ (Th1) as compared to the fusion alone. It should be considered that the high levels of Th1 could be effective for the eradication of intracellular reservoirs of UPEC in the bladder. It is also possible that there was a correlation between the production of IL.17 and IFN.γ, since secretion of IL.17 has helped to induce the high titer of IFN.γ by the vaccine combinations^33,34^. Unlike some studies indicating mucosal antibody responses were not increased in subcutaneous immunization^35^, our study demonstrated that mucosal IgG response was induced in the subcutaneous route of administration. Wiser et al. also reported that the use of different epitope rich domains in multi-epitope vaccines from subcutaneous route had the potential to induce IgA responses^15^.

In the bladder challenge, we found that all vaccinated mice significantly reduced bacterial levels in the bladder two days after bladder infection, with a trend towards the reduction of bacterial load in kidneys. Among the candidates, the fusion admixed with Freund showed the highest protection in the bladder and kidneys against *E. coli*. Interestingly, this vaccine combination revealed the full protection against the kidney infection in 40% of the immunized mice after the first and second challenge assay. It was found that the humoral and cellular (IFN.γ and IL.17) responses induced in the full protected mice (2 of 5 mice) were in the highest levels compared to the other immunized mice. Therefore, the high levels of protection among the mice could be attributed to the humoral (mucosal or systemic) or cellular responses or a mixture of both^33,36^. There may also be a direct relation between the decrease of UPEC levels recovered from the bladders and decrease of infection in the kidneys, thus the reduction of UPEC infection in the bladder may have resulted in the kidney protection.

Memory B cells are essential in the prevention against UTIs, especially recurrent UTIs^13^. In our study, the immunized groups with and without Freund maintained high levels of the IgG response until 6 months after the first vaccine dose. In addition, the repetition of the mouse challenge model after 6 months post first immunization confirmed the efficacy of sustained responses in the protection of urinary tract against UPEC. This finding was one of the prominent features of our designed vaccine candidate which could be effective in the eradication of bacterial reservoirs for a long time.

Our study has a limitation. This study was the first step of developing a novel vaccine against UTIs caused by UPECs. In this step we assessed the efficacy of the vaccine only with UPEC strain CFT073 and it was not possible to evaluate its efficacy in mice with clinical UPEC strains. We are going to evaluate the efficacy of this vaccine with different UPEC strains in the future.

In the present study, we designed and constructed a novel multi-epitope vaccine candidate from UPEC strain based on in silico methods. This study showed the potential of the candidate in the induction of immune responses, and the protection of urinary tract against UPEC. Therefore, our designed candidate could be a promising candidate against UTIs caused by UPEC which requires further investigations. Our results could also be useful in gaining insight towards the potential of epitope-based construct as an important protective and therapeutic approach for bacterial immunization.

## Methods

### Ethics statement

The animal and human studies were performed according to the ethical safety guidelines of Pasteur Institute of Iran and confirmed by the Ethical Committee of the Pasteur Institute of Iran under Ethical Number: IR.PII.REC.1395.73. Written informed consent to perform the humoral response in serum was obtained from all patients and normal human cases. Thus, all experiments were performed in accordance with relevant guidelines and regulations and we explicitly confirm that the studies were approved by the Ethical Committee of the Pasteur Institute of Iran.

### Primary sequence analysis and domain identification

IutA (Accession No. AAN82071.1) and FimH (Accession No. AAN83822.1) sequences from UPEC strain CFT073 were retrieved from National Centre for Biotechnology Information (NCBI) (www.ncbi.nlm.nih.gov). TM BETA software was used to identify the extracellular domains of proteins (https://psfs.cbrc.jp/tmbeta-net/).

### Immuno-informatics analyses

#### Prediction of linear, discontinuous, and antigenic B-cell epitopes

B-cell epitopes were predicted using BCPREDS (https://ailab.cs.iastate.edu/bcpreds/predict.html) and IEDB (https://www.iedb.org/) servers as described previously^37,38^. Discontinuous B-cell epitopes were designated from the refined and validated 3D protein structures by Ellipro at IEDB sever (https://tools.immuneepitope.org/toolsElliPro/)^39^.

#### Prediction of MHC-I binding epitopes

As A*0201 is the most common MHC-I bound allele in human^40^, this allele was chosen for epitope prediction. MHC-I binding epitopes in BALB/c mice were also considered in the prediction to have strong T cell responses in mice. 9mers epitopes were selected, because most HLA molecules have strong preferences for binding to 9mers^41^. Three different algorithms were employed for prediction of HLA-I epitopes, including SYFPEITHI (cut off score > 20)^42^, Net MHC 4.0 (an artificial neural network), and RANKPEP which utilize a position specific scoring matrix (PSSM) to predict MHC-I restricted epitopes^43^.

#### Prediction of MHC-II binding epitopes

Nine human HLA-II super type alleles, as well as Ad and Ed mouse alleles were chosen for MHC-II epitope prediction. IEDB server (https://tools.immuneepitope.org/mhcii/) was used to predict MHC-II epitopes using NetMHCII and Consensus methods^44^. RANKPEP and SYFPEITHI servers were used for identification of mouse MHC-II epitopes.

#### Antigenicity, allergenicity, and solubility assessments

The antigenicity of the designed sequence was assayed using VaxiJen v2.0 server, as described previously^45^. The allergenicity and solubility of the designed sequence were assessed using AlgPred (https://www.imtech.res.in/raghava/algpred/)^46^ and SOLpro (https://scratch.proteomics.ics.uci.edu)^47^, respectively. The physicochemical parameters of the designed protein such as molecular weight, number of positive and negative residues, half-life, and instability index were assessed by ProtParam (https://us.expasy.org/tools/protparam.html)^48^.

### Vaccine features

#### Protein's tertiary structure prediction, refinement, and validation

I-TASSER (https://zhanglab.ccmb.med.umich.edu/I-TASSER) that identifies similar structure templates from the Protein Data Bank (PDB) was used to predict the tertiary structure of the designed sequence^49^. Then, the best-modeled structure was refined by GalaxyRefine (https://galaxy.seoklab.org/cgi-bin/submit.cgi?type=REFINE)^50^. The refined model was finally analyzed using protein structure analysis (ProSa) (https://prosa.services.came.sbg.ac.at/prosa.php)^51^, Molprobity, and Ramachandran Plot Analysis resource (RAMPAGE)^52^.

### Molecular interaction studies of TLR4-protein vaccine

#### Molecular docking

The 3D structure of TLR4 was extracted from Protein Data Bank (PDB: 3FXI). Then, molecular docking of TLR4 and protein vaccine was done using Cluspro2 software^53^. The docked complexes and interactions were analyzed and visualized using LigPlot + v.1.4.5.

#### Molecular dynamics simulations

The stability of the docked structures was evaluated by molecular dynamics (MD) simulations using GROMACS MD package 5.0.1 with Amber99sb-ildn force field^54^.

#### Analysis of amino acid sequences

The amino acid sequences of FimH and IutA used for construction of the poly-epitope were aligned with the sequences of UPEC strains available in NCBI and analyzed with BLAST (www.ncbi.nih.gov) tool.

#### Codon optimization and cloning of the designed gene

The designed construct was translated reversely to DNA sequence. After codon optimization and adding a 6 His-tag to C-terminus of the sequence, the construct was cloned into pET28a expression vector (Novagen, USA) by Biomatik Company (Canada).

#### Expression and purification of the recombinant protein

The recombinant expression vector containing the designed sequence was transformed into *E. coli* BL21 (DE3) cells. Recombinant clones were selected by different methods such as Polymerase Chain Reaction (PCR), restriction enzyme digestion, and finally sequencing. Expression of the recombinant protein was induced by adding different concentrations of isopropyl-beta-thio galactopyranoside (IPTG) and assayed using SDS-PAGE and Western blot analysis using His tag monoclonal antibody (Sigma, USA) at dilution of 1:1000. The expressed protein was then purified using His tag affinity chromatography on Ni–NTA column (Qiagen), according to the protocol. Limulus amebocyte lysate (LAL) test was performed to determine the level of LPS contamination. Finally, the purified protein was dialyzed overnight and quantified using Bradford method.

#### Assessment of IgG responses in human

After purification of the recombinant protein, possible IgG response against the protein was assessed in serum of previously UPEC-infected people (n = 10). An ELISA procedure was used for this, testing serial serum dilution from 1:10 to 1:1000 as described in the next section. Human sera from non-infected people were used as negative control.

#### Mice immunization

Four groups of BALB/c mice (n = 15) were subcutaneously inoculated with 50 μg of fusion protein alone, fusion protein mixed with Freund adjuvant (50 μg protein + 50 μl adjuvant), or PBS, and Freund adjuvant alone (control groups) on days 0, 14, and 28. Sera were collected on days 15, 30, 45, 75, 110, 145, and 180 post first inoculation. The urine was also collected 45 days after the first vaccine dose to determine the humoral responses.

#### Assessment of humoral immune responses in mice

The antibody responses including IgG, IgG1, IgG2a, and IgA were quantified by enzyme-linked immunosorbent assay (ELISA) as described previously^55,56^. Briefly, purified fusion protein at a concentration of 10 μg/ml (1 μg/well) was coated onto microtiter plates (Greiner, Germany), and incubated overnight at 4 °C. The boundless positions in plates were blocked with 3% bovine serum albumin (BSA) (Sigma, USA) for 2 h at 37 °C, followed by 4 times washing with PBS-T buffer (PBS1x + Tween 20) and incubated with serial dilutions of serum (1:100–1:6400) or urine samples (undiluted to 1:10) for 90 min at 37 °C. Finally, HRP-conjugated secondary antibodies and TMB substrate were applied into the wells and the absorbance was measured at a wavelength of 450 nm using an ELISA reader.

#### Cytokine assay

For cytokine assessment, mice (n = 5) were sacrificed 45 days after the first vaccination and the spleens were isolated and homogenized. Then, the homogenized splenocytes were cultured at a density of 3 × 10^5^ and stimulated with fusion protein (10 μg/ml) to collect the supernatants after 3 days. Finally, the levels of IFN.γ, IL.17, and IL.4 were measured by the DuoSet ELISA kit (R&D Systems, USA).

#### Bladder challenge assay in the immunized mice

Bladder challenge assay was performed on days 48 and 180 after the first vaccination to evaluate the efficacy and sustainability of developed immune responses. Five mice out of 15 immunized mice were used for the first challenge and the remaining 5 mice were kept until day 180 were used for the second challenge assay. In brief, *E. coli* strain CFT073 was introduced transurethrally by micro catheter (B&D, USA) into the bladder of anesthetized mice with a mixture of ketamine and xylazine (70 mg/kg + 5 mg/kg) (Alfasan, the Netherland). After one week, kidneys and bladders of the challenged mice were isolated, homogenized, and cultured in LB agar medium to count the appeared colonies.

#### Statistical analysis

The statistical SPSS software (SPSS package program version 19, IBM Corporation) was used for analysis of immune responses. All experiments were conducted in three independent experiments and results were expressed as average ± S.D. Analysis of variance (ANOVA) was used for statistical analysis of the immune responses including total IgG, IgG isotypes, IgA, and cytokine levels (IFN.γ, IL.4, and IL.17), followed by Tukey or Games-Howell tests. Comparison of IgG responses between the infected humans with UPEC with humans that had no history of infection with UPEC was performed by Student t-test. Analysis of the challenge experiments was done using Prism program (GraphPad), version 6, with Kruskal–Wallis and Mann–Whitney. P value less than 0.05 was considered as a statistically significant difference between groups.

## Supplementary information

Supplementary Information.
