# Supplementary material for: In silico analysis and in vivo assessment of a novel epitope-based vaccine candidate against uropathogenic Escherichia coli
Source: Sci Rep. 2020 Oct 1;10:16258. doi: 10.1038/s41598-020-73179-w (PMC7530722; doi:10.1038/s41598-020-73179-w)
Supplement: Supplementary file 1 — Supplementary Information. [file 41598_2020_73179_MOESM1_ESM.docx]

***In Silico* analysis and *in vivo* assessment of a novel Epitope-Based Vaccine Candidate against Uropathogenic *Escherichia coli***

Sara Hasanzadeh^1^, Mehri Habibi^1^, Mohammad Ali Shokrgozar^2^, Reza Ahangari Cohan^3^, Khadijeh Ahmadi^4^, Mohammad Reza Asadi Karam^1*^, Saeid Bouzari^1*^

^1^Department of Molecular Biology, Pasteur Institute of Iran, Tehran, Iran. ^2^National Cell Bank, Pasteur Institute of Iran, Tehran, Iran. ^3^Department of Nanobiotechnology, Pasteur Institute of Iran, Tehran, Iran. ^4^Professor Alborzi Clinical Microbiology Center, Shiraz University of Medical Sciences, Shiraz, Iran. Correspondence and requests for materials should be addressed to S.B. (email: [saeidbouzari@yahoo.com](mailto:saeidbouzari@yahoo.com)) and M.R.A.K. (email: m_asadi12@yahoo.com)

**Supplementary Table S1.** Prediction of MHC-I epitopes from full-length protein IutA.

| **Server** | **Amino acid position** | **Score or rank** |
| --- | --- | --- |
| Syfpeithi | 98-106  169-176  257-265  313-321  446-454  609-617  749-757 | 31 (score)  24  23  20  20  23  21 |
| Net MHC | 97-105  445-453  499-507  579-587  696-704  748-756 | 0.15 (% Rank)  101  0.4  1.6  0.2  0.4 |
| Rankpep/ Cleavage site | 98-104  156-164  289-297  433-442  500-508  609-617  707-715 | 78/ + (Score)  68/ +  75/ +  66/ +  68/ +  67/ -  73/ - |
| Syfpeithi Mouse | 430-438  440-448  499-507  459-467  585-593  733-741 | 25 (Score)  20  21  22  23  23 |
| Net MHC Mouse | 215-223  429-437  458-466  511-518  545-553  579-588  584-592  653-661 | 0.7 (% Rank)  0.2  0.1  0.07  0.25  0.4  0.09  0.5 |
| Rankpep Mouse/ Cleavage site | 147-155  430-438  526-534 | 25/ + (Score)  31/ +  25.6/ - |

**Supplementary Table S2.** Prediction of MHC-II epitopes from full-length protein IutA.

| **Server** | **Amino acid position** | **Score or Rank** |
| --- | --- | --- |
| IEDB Human | 144-158  267-281  343-357  371-385  499-513  512-526  581-595  599-613  668-682 | 0.43 (% Rank)  0.29  0.97  1.61  1.72  0.29  0.77  0.52  0.68 |
| Net MHC Human | 124-138  335-349  404-418  580-594  598-612 | 2 (% Rank)  1.6  1.6  0.7  1.7 |
| Syfpeithi Mouse | 149-163  202-216  349-363  355-369  429-443  473-487  481-495  546-560  576-590  585-599  598-612  673-687 | 23 (Score)  27  21  26  23  28  25  32  26  22  29  22 |
| Rankpep Mouse | 292-300  477-485  522-530  535-543  560-568  724-732 | 11.9 (Score)  13.9  12.1  8.5  7.6  10 |
| NET MHC Mouse | 287-300  431-445  472-491  513-530  530-549 | 0.9 (% Rank)  1.7  0.7  1.3  1 |

**Supplementary Table S3.** Prediction of MHC-I epitopes from full-length protein FimH.

| **Servers** | **Start position** | **End position** | **The Best ranked epitope in the region** |
| --- | --- | --- | --- |
| Syfpeithi (Human) | 33  48  88  141  185  192  217  251  263  284 | 41  56  96  150  193  200  225  259  270  292 | 21 (Score)  20  88  22  23  24  22  25  20  23 |
| Net MHC  (Human) | 87  162 | 95  170 | 0.7 (% Score)  0.12 |
| Rankpep/Cleavage site  (Human) | 55  88  188  192  217  228  245  251  263 | 63  96  193  200  225  236  253  259  271 | 86/ - (% Score)  88/ +  64/ -  67/ -  66/ -  65/ +  65/ +  69/ +  80/ + |
| Syfpeithi | 41  122  128  144  196  215  253  276 | 49  130  136  152  204  223  261  284 | 23 (Score)  24  25  20  22  23  23  22 |
| Net MHC | 33  40  83  96  121  127  195  205  209  214  252  263 | 41  48  91  104  129  135  203  303  217  222  261  275 | 0.17 (% Rank)  0.4  0.8  0.6  0.06  0.25  0.04  0.4  0.5  0.5  0.08  0.08 |
| Rankpep/Cleavage site | 41  169  196  276 | 49  177  204  284 | 15.5/ + (Score)  17.6/ -  23.7/ +  16.6/ - |

**Supplementary Table S4.** Prediction of MHC-II epitopes from full-length protein FimH.

| **Server** | **Position** | **Score or Rank** |
| --- | --- | --- |
| IEDB Human | 36-54  49-67  70-90  79-97  108-126  124-138  141-160  157-177  161-177  211-229 | 0.78 (Rank)  0.13  0.23  0.8  1.15  1.7  0.48  1.08  0.65  0.98 |
| Net MHC Human | 36-53  49-66 | 0.8 (% Rank)  1.3 |
| Syfpeithi Mouse | 35-49  40-34  71-85  82-104  107-121  135-149  140-154  169-183  198-212  217-231 | 32 (Score)  25  26  24  21  22  25  22  24  26 |
| Rankpep Mouse | 42-50  62-70  99-107  164-172  205-213  280-288 | 8.9 (Rank)  7.6  14  12.8  7.9  7.1 |
| Net MHC Mouse | 36-53  122-141  210-239 | 0.5 (% Rank)  1  0.7 |

1.
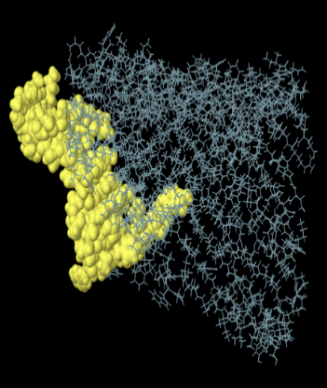

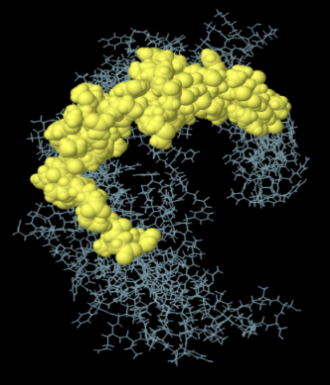
 (B) (C)


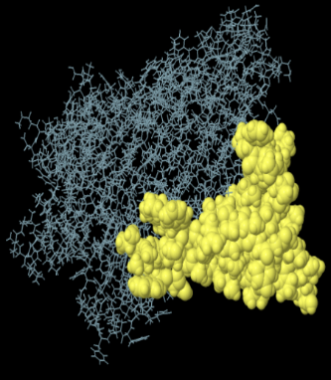


**Supplementary Figure S1.** 3D Representation of the predicted discontinuous epitopes of the final designed protein (A to C).


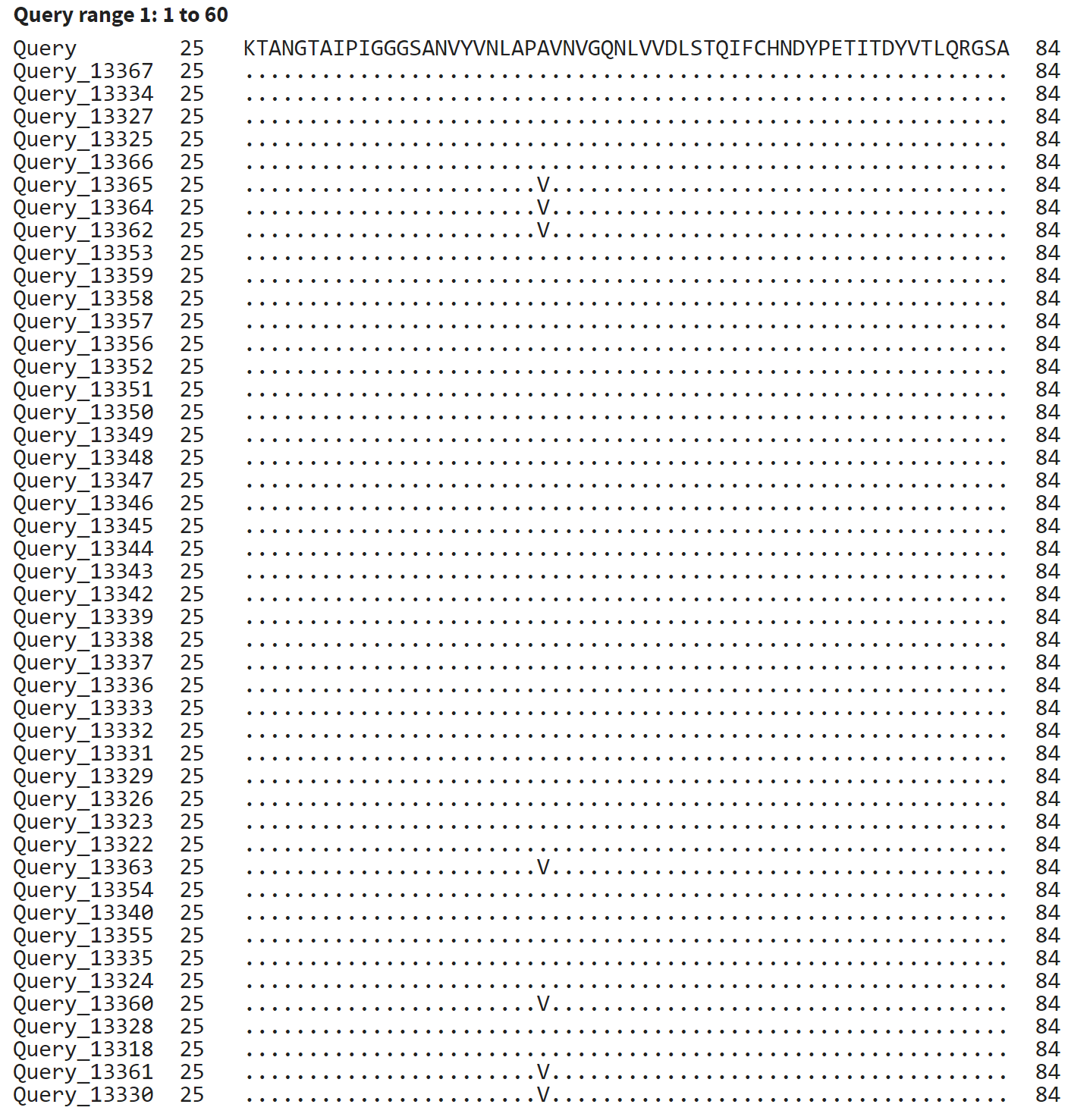

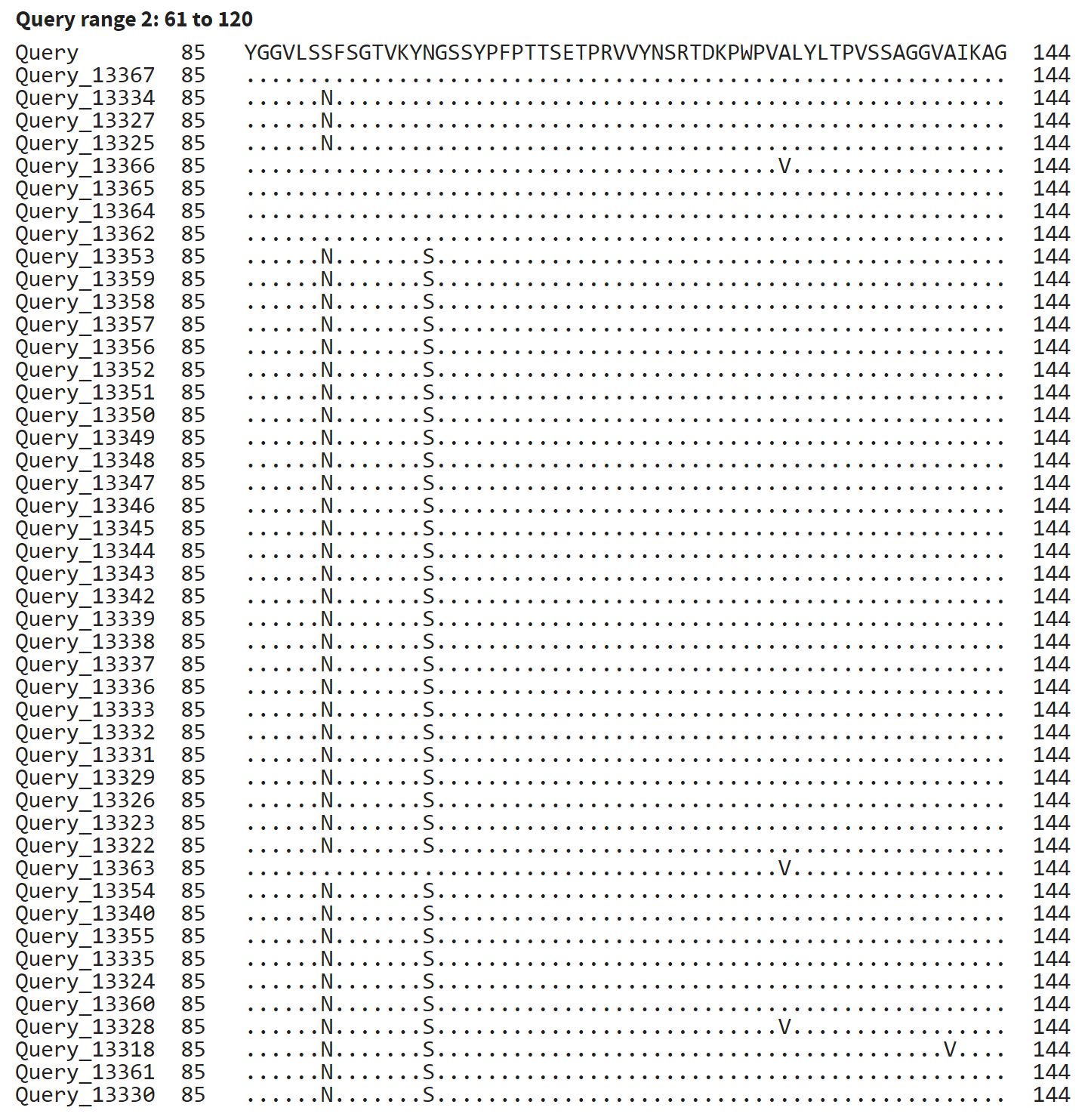


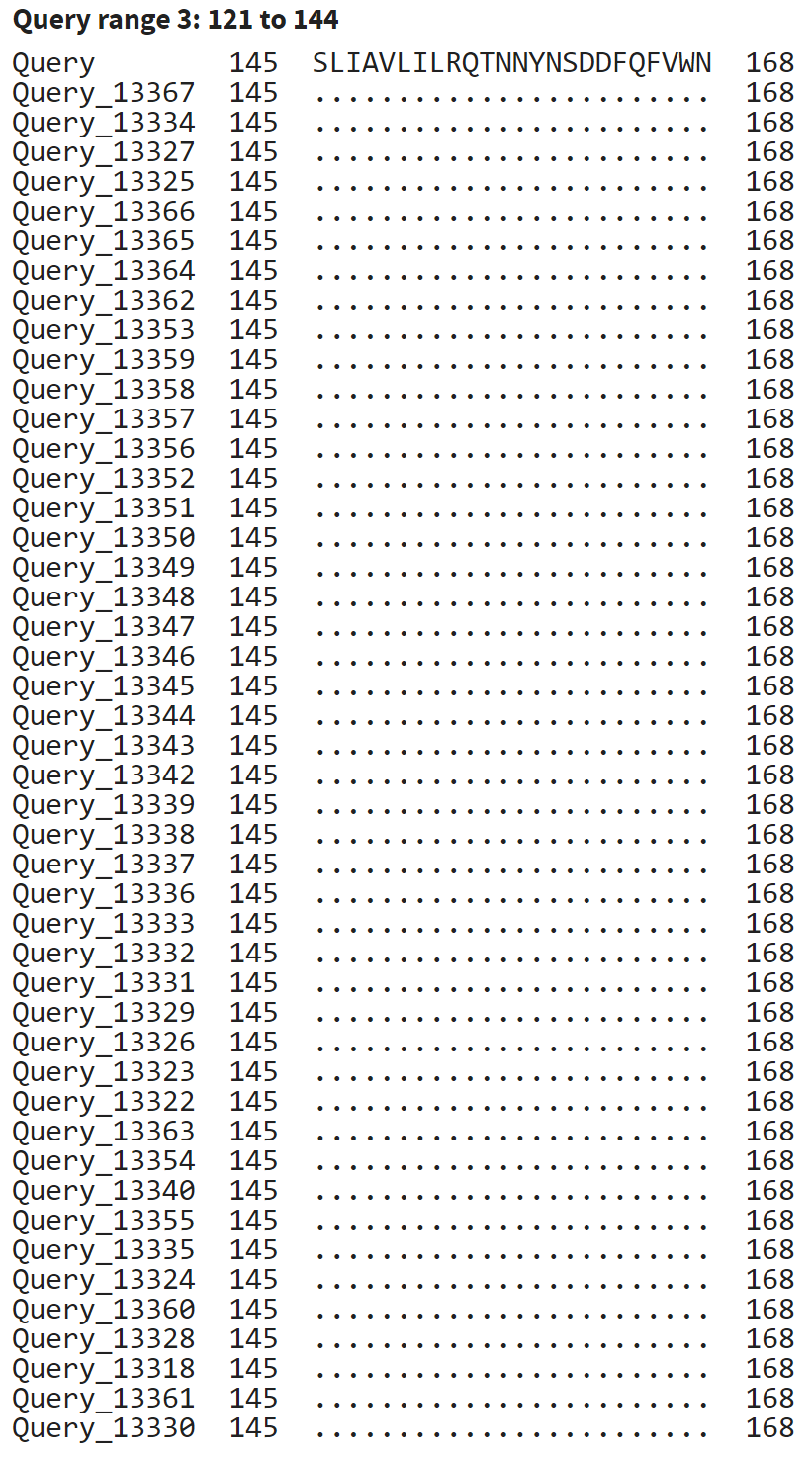


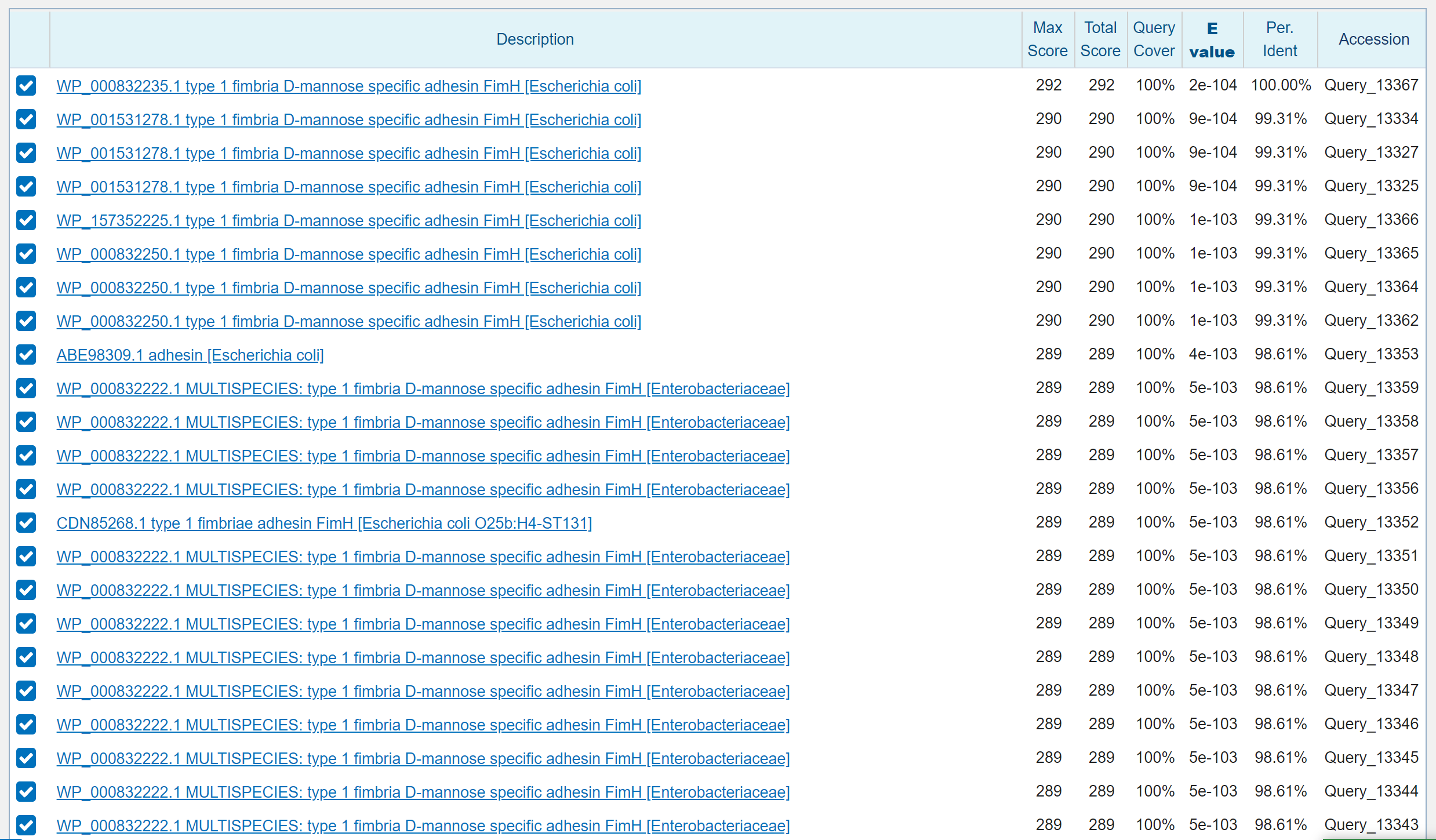

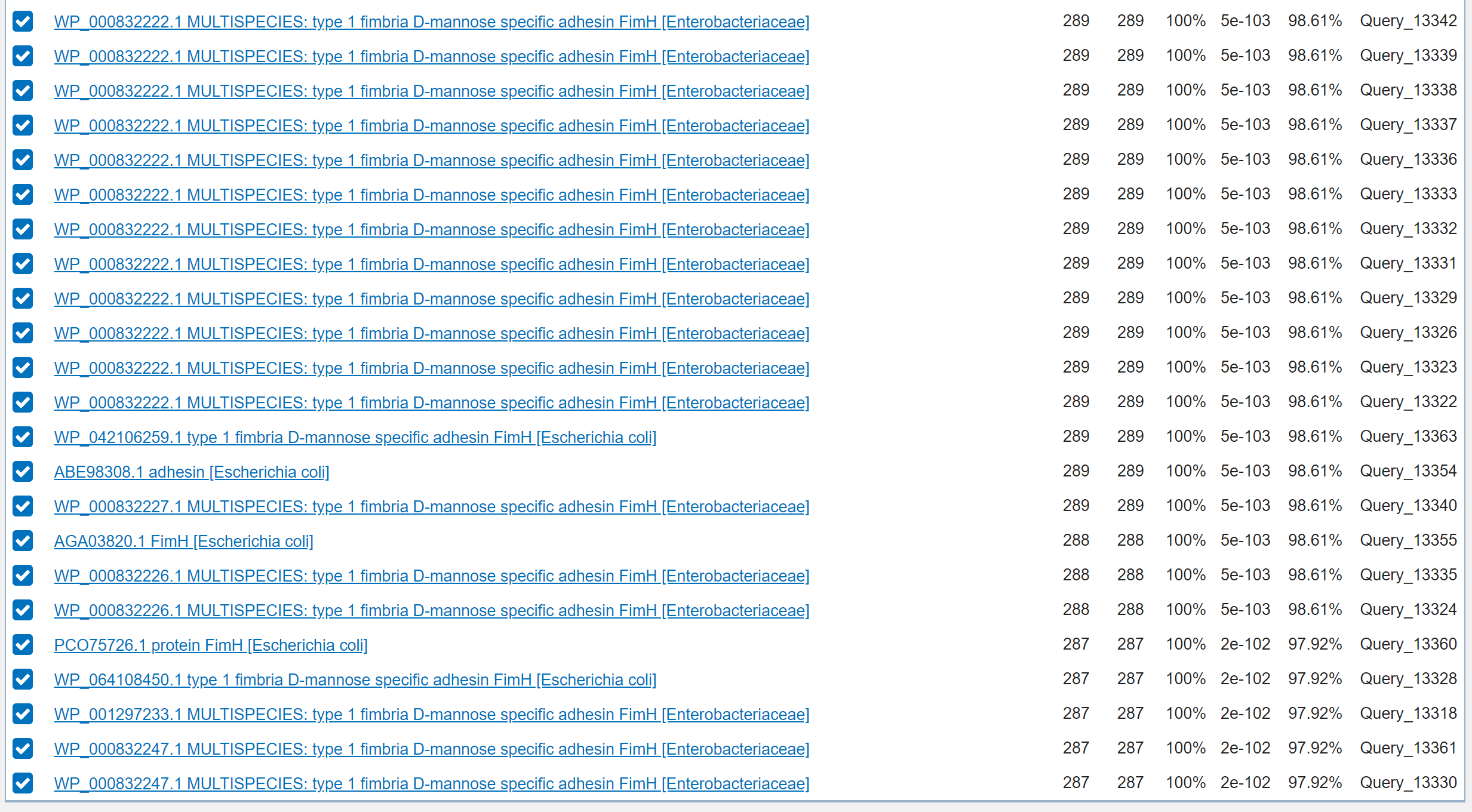


**Supplementary Figure S2.** Multiple alignments of amino acid sequences of FimH used for construction of the poly-epitope with sequences of FimH available in NCBI.


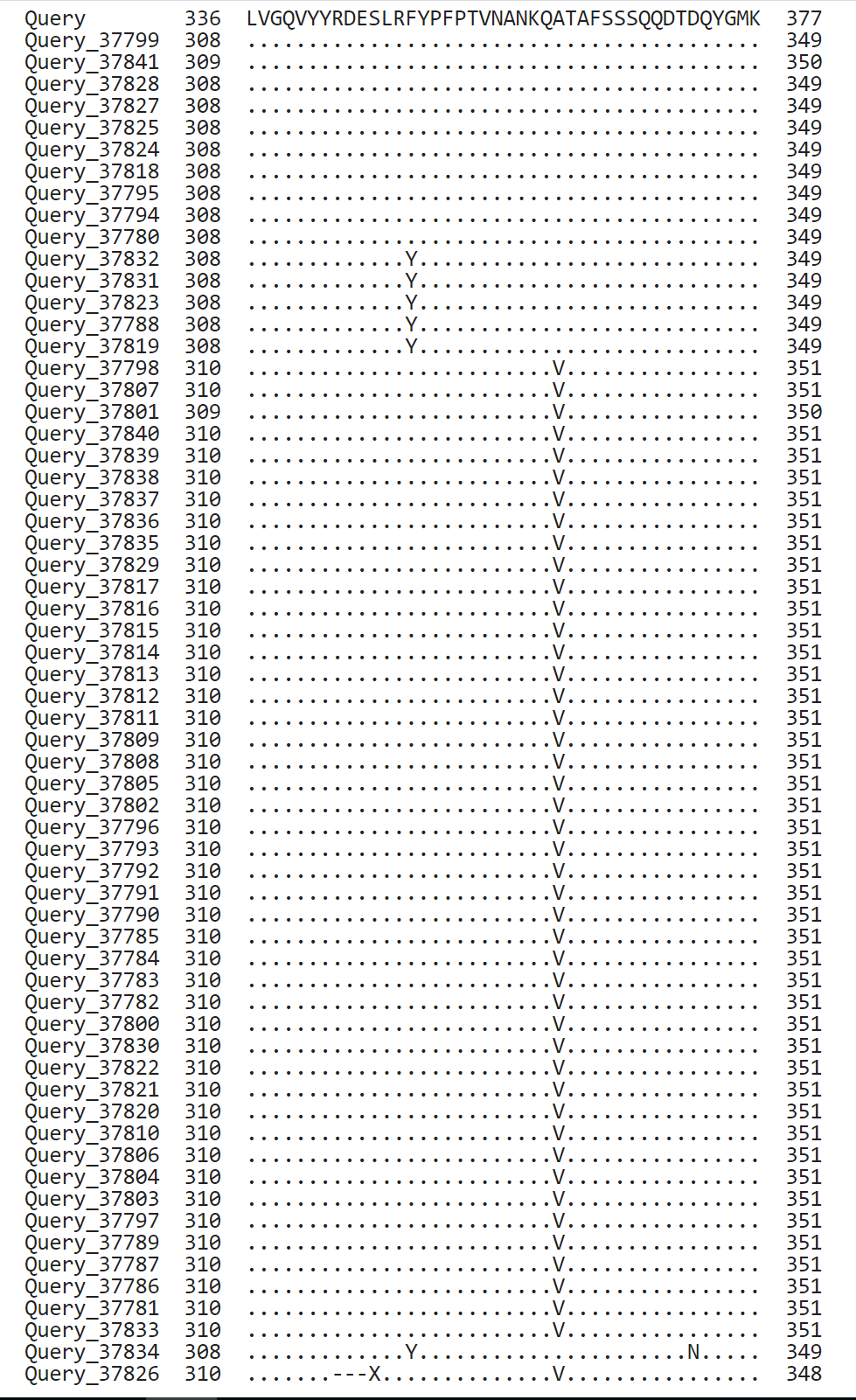

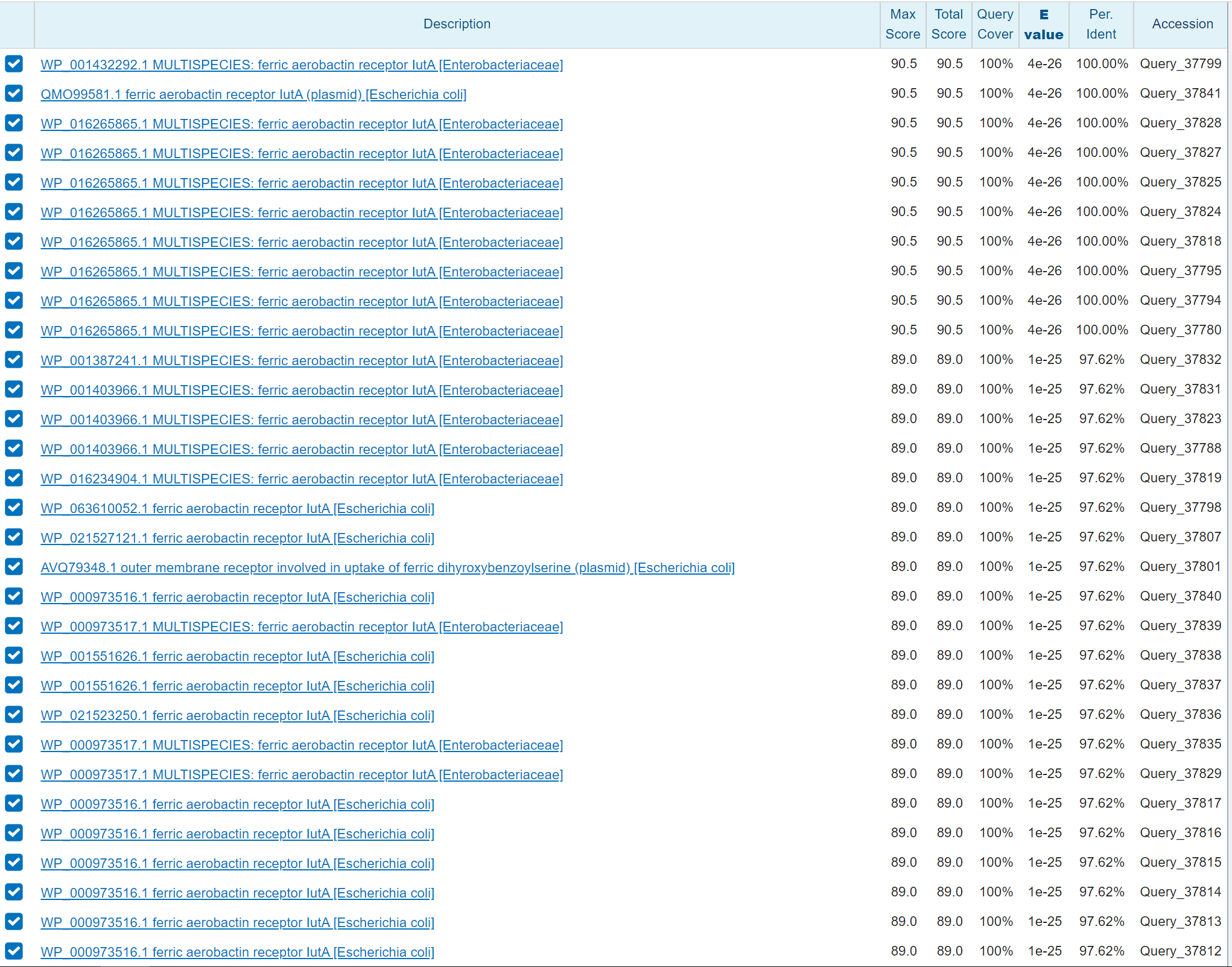

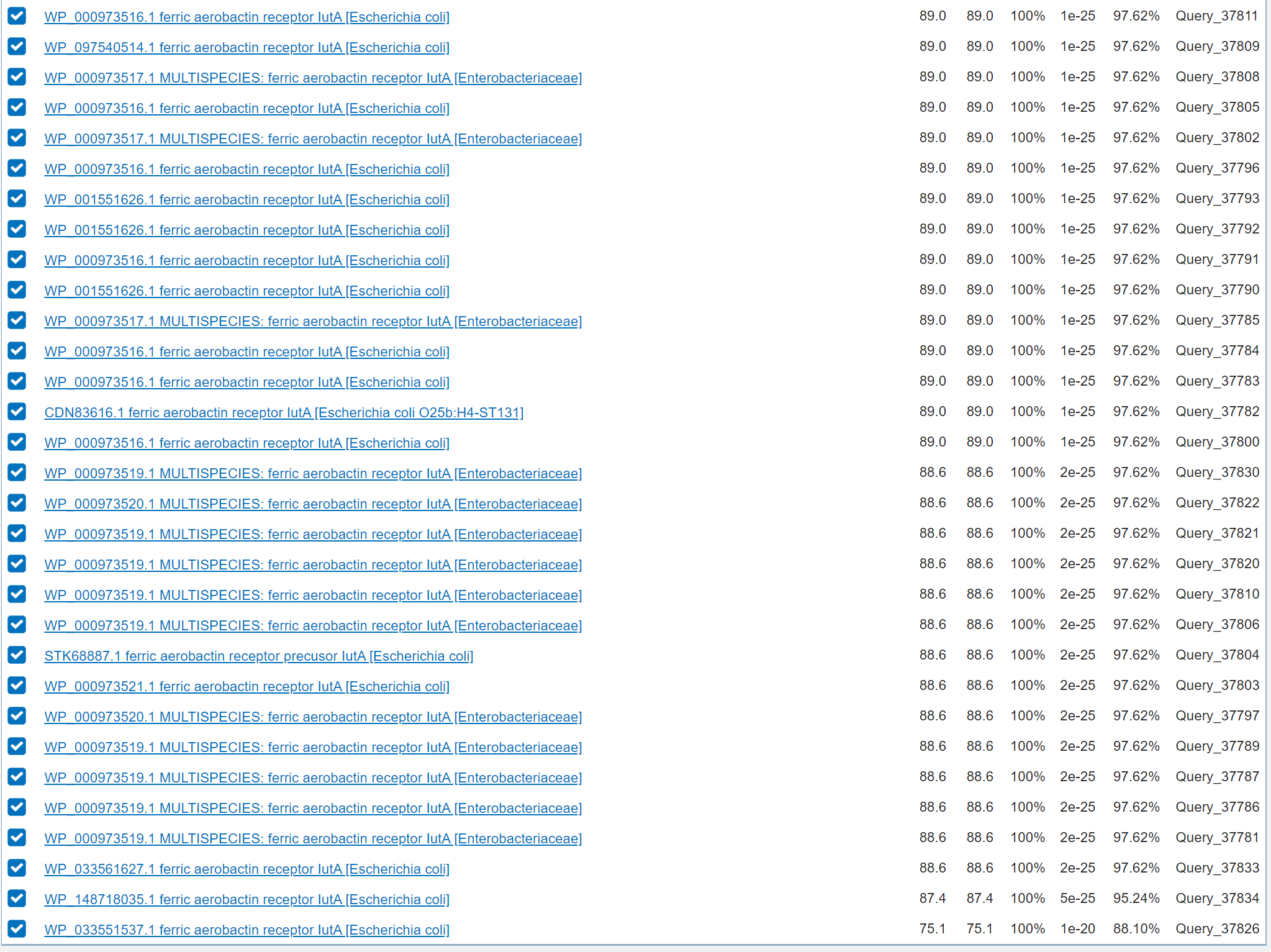


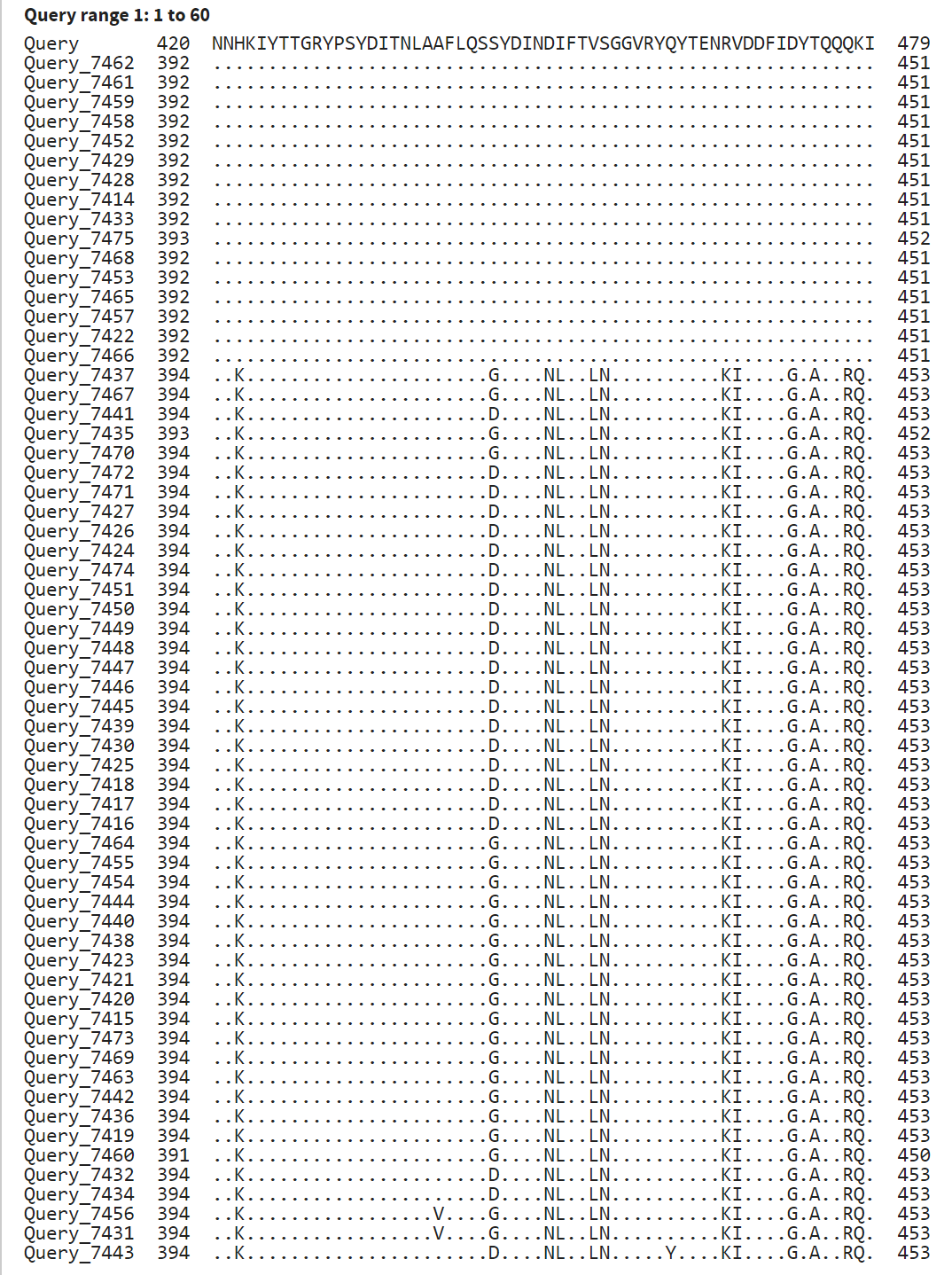

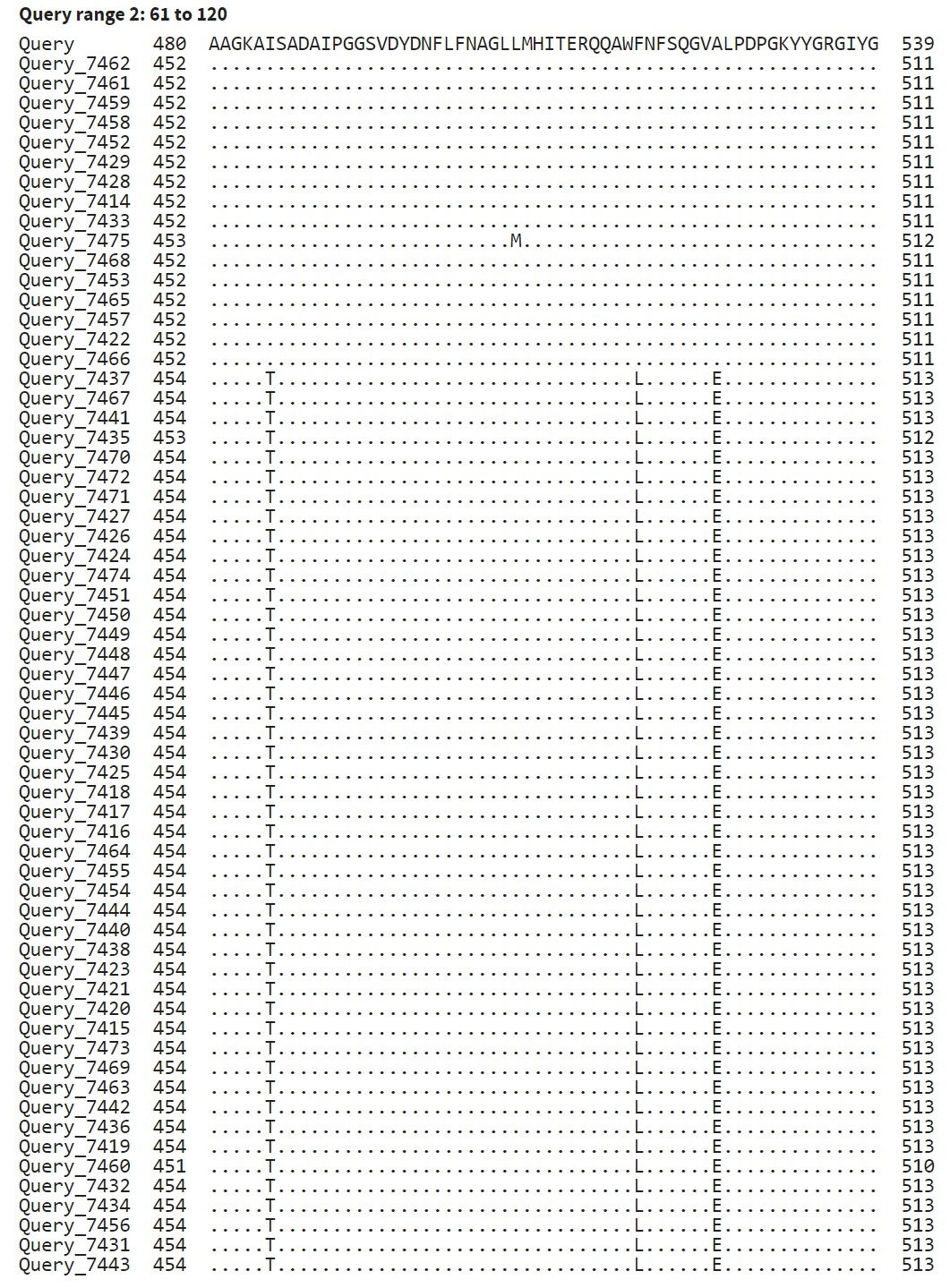

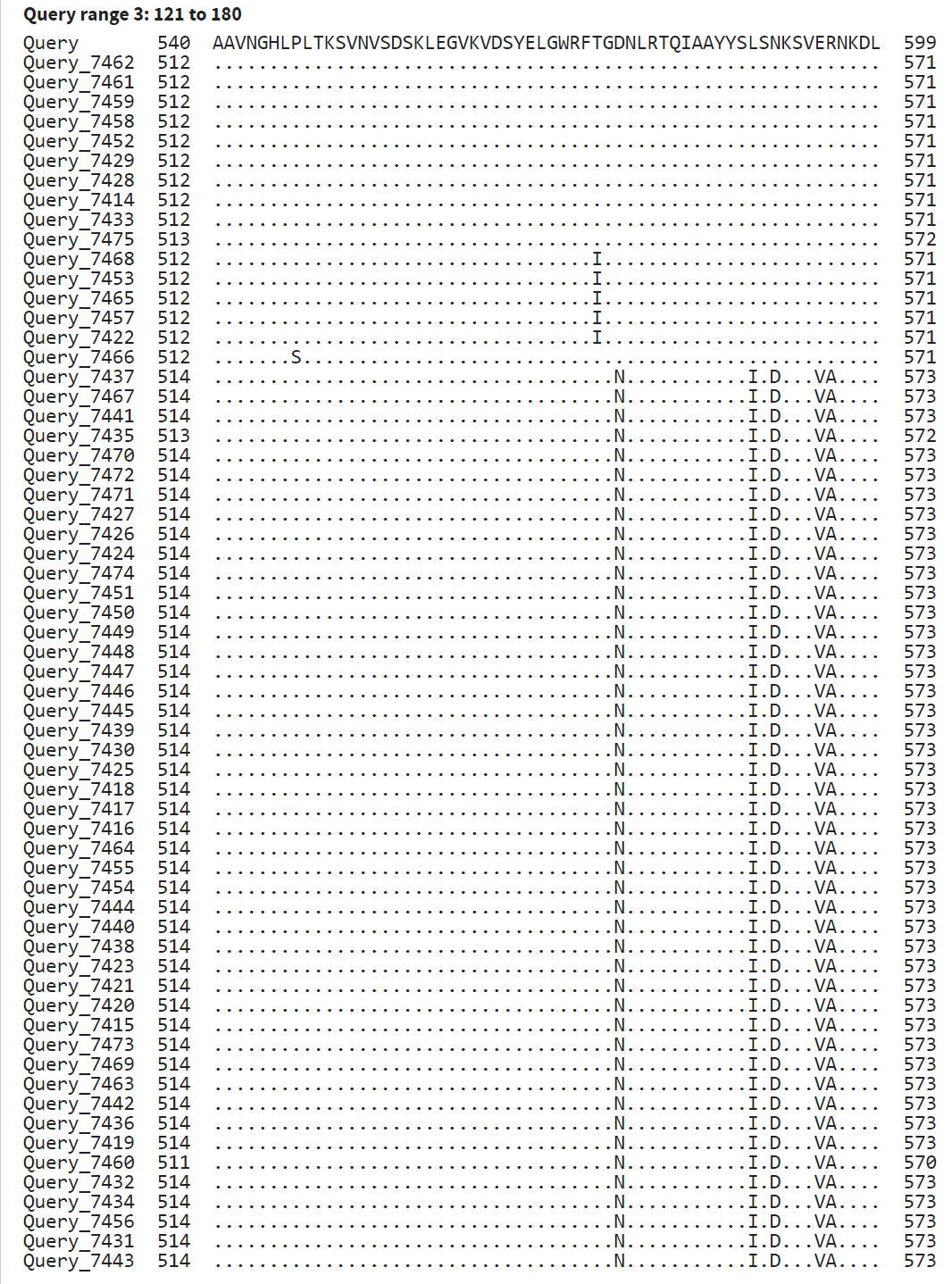


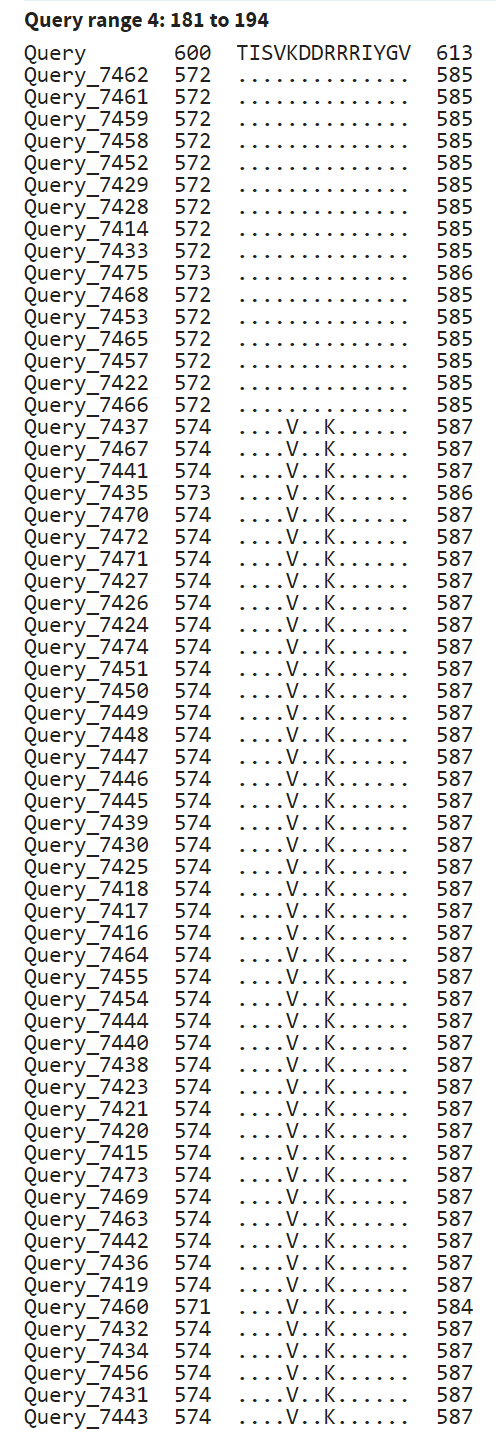


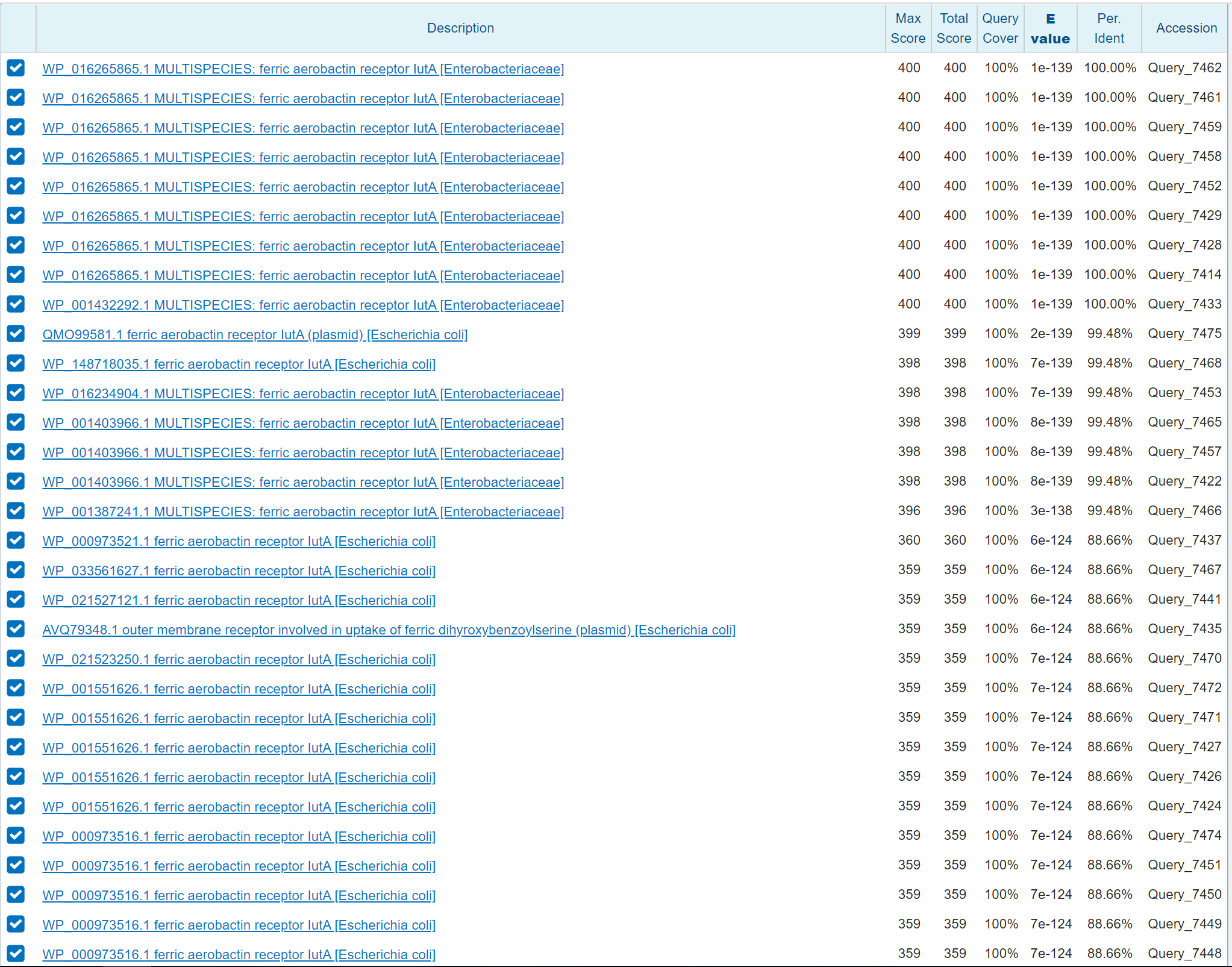

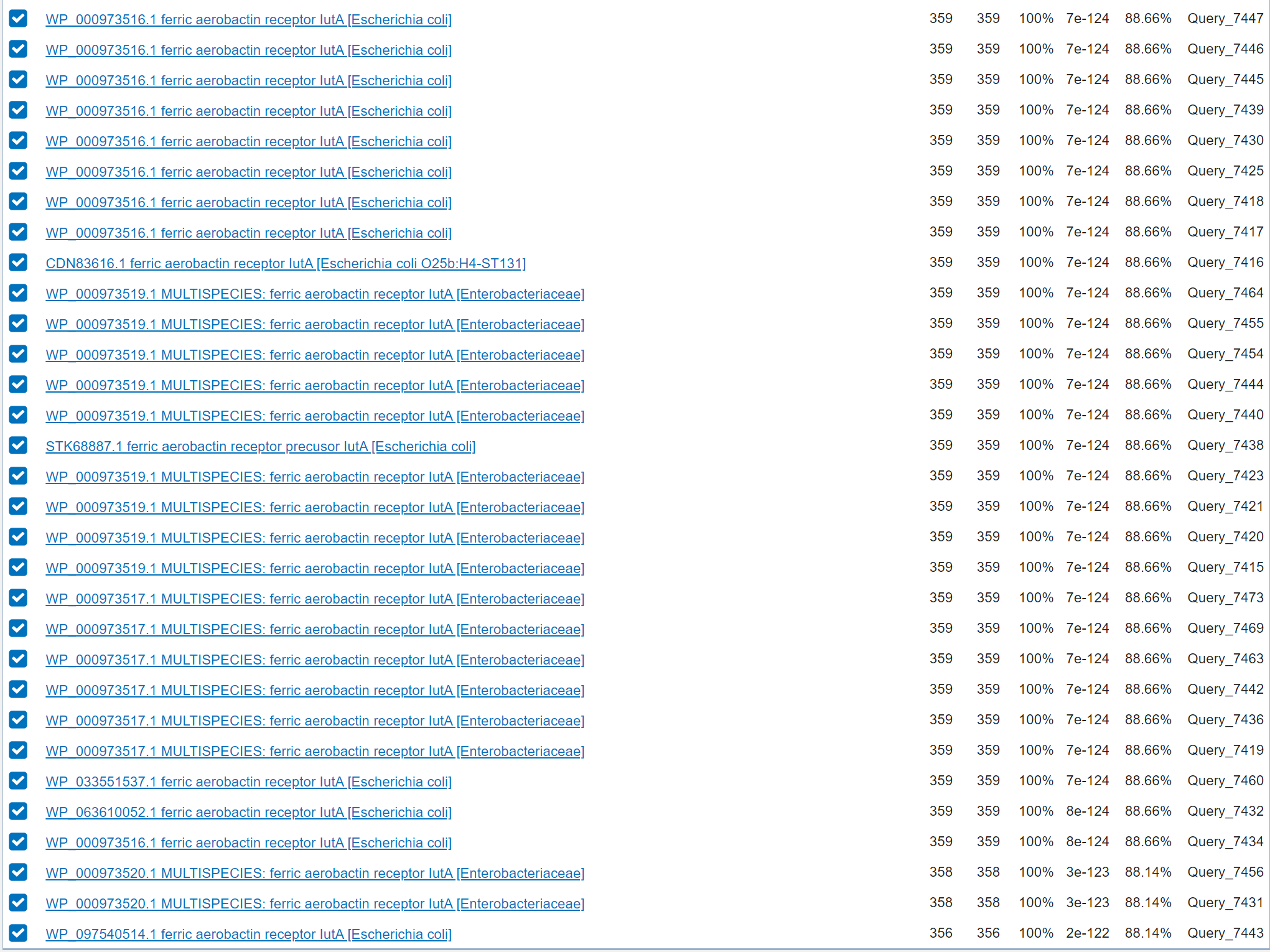


**Supplementary Figure S3.** Multiple alignments of amino acid sequences of IutA used for construction of the poly-epitope with sequences of IutA available in NCBI.
